# Adaptive LASSO estimation for functional hidden dynamic geostatistical models

**DOI:** 10.1007/s00477-023-02466-5

**Published:** 2023-05-17

**Authors:** Paolo Maranzano, Philipp Otto, Alessandro Fassò

**Affiliations:** 1 Department of Economics, Management and Statistics (DEMS), University of Milano-Bicocca, Piazza dell’Ateneo Nuovo 1, 20126 Milano, Italy; 2 Fondazione Eni Enrico Mattei (FEEM), Corso Magenta 63, 20123 Milano, Italy; 3 Insitute of Cartography and Geoinformatics (IKG), Leibniz University of Hannover, Appelstrasse 9a, 30167 Hannover, Lower Saxony Germany; 4 Department of Economics, University of Bergamo, Via dei Caniana 2, 24127 Bergamo, Italy

**Keywords:** Functional HDGM, Adaptive LASSO, Model selection, Penalized maximum likelihood, Geostatistical models, Air quality Lombardy

## Abstract

We propose a novel model selection algorithm based on a penalized maximum likelihood estimator (PMLE) for functional hidden dynamic geostatistical models (f-HDGM). These models employ a classic mixed-effect regression structure with embedded spatiotemporal dynamics to model georeferenced data observed in a functional domain. Thus, the regression coefficients are functions. The algorithm simultaneously selects the relevant spline basis functions and regressors that are used to model the fixed effects. In this way, it automatically shrinks to zero irrelevant parts of the functional coefficients or the entire function for an irrelevant regressor. The algorithm is based on an adaptive LASSO penalty function, with weights obtained by the unpenalised f-HDGM maximum likelihood estimators. The computational burden of maximisation is drastically reduced by a local quadratic approximation of the log-likelihood. A Monte Carlo simulation study provides insight in prediction ability and parameter estimate precision, considering increasing spatiotemporal dependence and cross-correlations among predictors. Further, the algorithm behaviour is investigated when modelling air quality functional data with several weather and land cover covariates. Within this application, we also explore some scalability properties of our algorithm. Both simulations and empirical results show that the prediction ability of the penalised estimates are equivalent to those provided by the maximum likelihood estimates. However, adopting the so-called one-standard-error rule, we obtain estimates closer to the real ones, as well as simpler and more interpretable models.

**Supplementary Information:**

The online version contains supplementary material available at 10.1007/s00477-023-02466-5.

## Introduction

In recent years, we experienced a significant increase in the availability and size of geo-referenced datasets, especially in air quality monitoring (Vitolo et al. 2016), agriculture and livestock farming (Fass‘o, A., Rodeschini, J., Moro, A.F., Shaboviq, Q., Maranzano, P., Cameletti, M., Otto, P. 2023; Brown et al. 2023), and climate (Cruz-Alonso et al. 2023; Czernecki et al. 2020). As a result, geostatistical applications need efficient algorithms for variable selection, that is for selecting relevant predictors from a large set of candidates.

In addition, geostatistical data often defined on a functional domain because of their characteristics, e.g., vertical atmospheric profiles in climate studies (Fassò et al. 2018) or off-shore coastal profile measurements for beach monitoring (Otto et al. 2021). A functional data approach may also be used to reduce the dimensionality of high-frequency temporal observations. For example, Ignaccolo et al. (2008) considered the time series of air quality measurements at many stations as functional observations. Also, to understand the bike-sharing system, Piter et al. (2022) considered daily 5-min usage profiles of a bike-sharing system as daily functional observations. Due to the spatial nature of the underlying process, further applications can be found in environmetrics (e.g., Franco-Villoria and Ignaccolo 2017; Ignaccolo et al. 2013; Giraldo et al. 2011), medicine (e.g., Aristizabal et al. 2019), econometrics (e.g., Pineda-Ríos et al. 2019). As a result, the above mentioned need for efficient variable selection should cover also functional data models.

### The *Big-*N*-problem*

In this context, statistical methods have to face the so-called *Big-*N*-problem*, in which the time complexity of estimation algorithms grows polynomially with an order greater than 2 when the number of locations is increasing and traditional methods are often not computationally feasible (cf. Katzfuss 2017a; Katzfuss and Cressie 2011). To reduce the complexity of such models, various approaches have been used, some of which are based on inducing sparsity in the spatial covariance matrix (Furrer et al. 2006; Kaufman et al. 2008; Stein 2013; Furrer et al. 2016). Some other approaches are related to the precision matrix, either using a graphical least absolute shrinkage and selection operator (LASSO) algorithm (Krock et al. 2021, 2021), or a sparse Cholesky factors approach based on the Vecchia approximations (Stein et al. 2004; Kang and Katzfuss 2021; Schäfer et al. 2021) and on multi-resolution approximations of Gaussian processes (Katzfuss 2017b; Jurek and Katzfuss 2021). In particular, Vecchia approximation can be efficently used to peform high-dimensional spatiotemporal filtering (Jurek and Katzfuss 2022) and spatiotemporal smoothing (Jurek and Katzfuss 2022b). Low-rank covariance matrices have been also considered, including fixed-rank kriging and penalised methods (Banerjee et al. 2008; Cressie and Johannesson 2008; Chang et al. 2010a; Hsu et al. 2012a; Cressie et al. 2010). Eventually, combined approaches, like the so-called *full-scale approximation of the covariance matrix* have been proposed (Sang and Huang 2012).

### Geostatistical variable selection

For large regression models, joint estimation and variable selection based on penalised estimation provides stable solutions compared to the classic backward and forward selection methods (Breiman 1996; Bondell et al. 2010). In this regard, we refer to the review paper of Müller and Welsh (2010) .

Methods of selecting covariates have been developed based on penalised methods in spatial and spatiotemporal settings. For instance, Wang and Zhu (2009) suggested a penalised least squares approach for spatial regression models; Cai and Maiti (2020), for spatial autoregressive models; and Gonella et al. (2022), for conditional autoregressive models. For additive spatial models including potential nonlinear effects, Nandy et al. (2017) developed a weighted penalised least squares estimator. Alternatively, penalised maximum likelihood estimators (PMLE) are considered. For instance, Zhu et al. (2010) suggested PMLE for linear models with spatially correlated errors. Chu et al. (2011a) and Chu et al. (2011b) additionally reduced the model’s complexity by combining covariance tapering and a PMLE for spatial and spatiotemporal settings, respectively.

It is important to note that geostatistical applications are prone to cross-correlated regressors due to their spatial nature. Cross-correlation among the regressors can be a critical issue in model selection. Indeed, when the covariates are correlated, as pointed out by Zhao and Yu (2006), the classic LASSO approach would generally not be selection-consistent. Similarly, Simon and Tibshirani (2012) showed that in the same situation, the group-LASSO estimator (cf. Yuan and Lin (2006)), which assumes orthonormal data within each group, would perform poorly in selecting the relevant features. Such issues motivate the choice of an adaptive LASSO penalty, which led to selection-consistent estimators in the case of cross-correlated regressors (see Zou 2006; Zou and Li 2008).

Following the least absolute shrinkage and selector operator (LASSO) methodology (Tibshirani 1996; Reyes et al. 2012) proposed a spatiotemporal adaptive LASSO algorithm for linear regression models with spatiotemporal neighbourhood structures. The estimation strategy involved both the penalised least squares and PMLE approaches. Other examples of penalised regression for spatiotemporal data are in Al-Sulami et al. (2019), in which an adaptive LASSO method was developed to simultaneously identify and estimate spatiotemporal lag interactions in the context of a data-driven semiparametric nonlinear model. Furthermore, Safikhani et al. (2020) considered LASSO methods for generalised spatiotemporal autoregressive models. The estimators are obtained by a modified version of the penalised least squares that accommodates hierarchical group LASSO-type penalties.

In general, spline basis functions are widely used tools in geostatistics for the spatiotemporal interpolation of environmental phenomena (see for group-LASSO approaches in this context Hofierka et al. 2002; Xiao et al. 2016; Chang et al. 2010b; Hsu et al. 2012b). Also, spatial and spatiotemporal model selection has been addressed in a Bayesian framework (see e.g., Katzfuss and Cressie 2012; Carroll et al. 2016, 2016; Lawson et al. 2017; Carroll et al. 2018) but it will not be the focus of this paper.

Penalised methods are also commonly applied in the context of functional data analysis, such as penalised splines (see Silverman and Ramsay 2002; Claeskens et al. 2009). These methods usually regularise the smoothness of the estimated functions by penalising the integrated second derivatives. In this way, many basis functions can be used, thus avoiding the typical overfit resulting from unpenalised estimation methods.

In the context of functional data, some authors have proposed the use of penalty methods for selecting relevant predictors. As an example, Pannu and Billor (2017) proposed using group LASSO for selecting grouped variables (functional predictors) rather than individual variables; Ivanoff et al. (2016) proposed adaptive LASSO and group LASSO estimators for functional Poisson regression; Zhao et al. (2012) employed a wavelet-based LASSO approach for regressing scalars on functions aimed at identifying a relatively small number of non-zero wavelet coefficients; eventually, Centofanti et al. (2022) proposed a smooth LASSO able to locate regions where the coefficient function is zero, and to smoothly estimate non-zero values of the coefficient function. These contributions, although close to us in terms of purpose and methodology, do not consider the presence of spatiotemporal dependence and cross-correlation between covariates, which are the main focus of this paper.

### Variable selection for spatiotemporal functional models

In this paper, we propose a penalised maximum likelihood estimate for the functional spatiotemporal model known as the *functional hidden dynamics geostatistical model* (f-HDGM) (Wang et al. 2021). More precisely, we develop an adaptive LASSO method able to estimate the relevant model coefficients and shrink to zero the irrelevant ones, while taking into account spatiotemporal correlation and cross-correlation among predictors.

In our functional setting, the LASSO selected coefficients are associated with the spline bases of the functional regressors. As a result we may have two cases. All the coefficients of a certain regressor are shrunk to zero, resulting in its drop out. The second and more subtle case, happens if only some of the coefficients of a single variable are shrunk to zero. In this case the functional coefficient may be zero in a subset of the functional domain. Similarly, when using periodic Fourier bases some frequencies for a specific variable may be neglected. For instance, Otto et al. (2021) showed that major storm floods have an effect only on specific parts of the coastal profiles, that is, those affected by high waves during a flood.

We test the performance of the algorithm through a Monte Carlo simulation study based on three settings with increasing level of complexity and representative of geostatistical applications. Furthermore, we apply the penalisation algorithm to an empirical example of air quality assessment. Within the application we study the computation time of the phases composing the penalty algorithm and its behaviour as the model complexity increases. Both simulations and applications are evaluated by highlighting the predictive ability of the penalised estimators, the interpretability of the estimates, the precision of parameter estimation, and the variable selection capability.

Our proposal extend the approach of Fassò et al. (2022), which considered a two stage variable selection algorithm approach for multivariate HDGM. In the first stage the classic trace LASSO is applied to the multivariate response variable without considering any spatiotemporal structure. This provides the so-called active sets associated to the penalty factors $\lambda$. In the second stage, for each active set the multivariate HDGM is estimated and tested using cross-validation. This provides the optimal active set and the corresponding selected variables according to an hybrid criterion. Here, considering functional response and predictors, we propose a penalized maximum likelihood method based on a second order approximation of the fHDGM likelihood.

The remainder of the paper is structured as follows. In Sect. 2, we briefly introduce the considered functional geostatistical model, namely the f-HDGM. In Sect. 3, we present the new penalised maximum likelihood approach using an adaptive LASSO penalty. In Sect. 4, we present the results of an extensive Monte Carlo simulation of three simulation settings. In Sect. 5, we illustrate an empirical application in which the penalisation algorithm is applied to daily air quality profiles in Lombardy, a region in Northern Italy. Finally, Sect. 6 concludes this paper and identifies potential topics for future research.

## The functional model

In this section, we review the functional spatiotemporal model known as the functional hidden dynamics geostatistical model. The modelling rationale may be rooted in the state-space modelling approach (Ferreira et al. 2022; Jurek and Katzfuss 2021, 2022b). It is based on the classic idea so that the temporal dynamics is described by a fixed effect component plus an unobserved Markovian component with innovations spatially correlated. In this framework, the spatiotemporal covariance is assumed to be separable (see for a comparison of different spatiotemporal models Huang et al. 2007). The multivariate model and its maximum likelihood estimation are introduced by Calculli et al. (2015). The procedure is based on maximising the likelihood function using an expectation-maximization (EM) algorithm, which is efficients thanks to the Kalman Filter algorithm (Rougier et al. 2023).

### Model details

The f-HDGM is designed to handle functional data $\{y_{s,t}(h): s \in D, t = 1, \ldots , T\}$ defined on the interval $H = [h_1,h_2]$. That is, $y_{s,t}(h): H \rightarrow \mathbb {R}$ can be observed at any $h \in H$ for any given location *s* in the spatial domain *D* and for any given discrete time *t*. Although the spatial domain *D* is continuous, we observe such data on *n* spatial points in an irregular grid $S=\{s_1,\ldots ,s_n\}$. Similarly, we observe the data for each function at a discrete set of points $h_1,\ldots ,h_q$, where both $h_i$ and *q* may depend on $s_i$ and *t*. These observations are denoted by vectors $\{y_{s,t}=(y_{s,t}(h_1),\ldots ,y_{s,t}(h_q)\}$. Overall, our data set is composed of $N = nT$ functional data.

To account for the spatial and temporal dependence, we model the process using a hidden dynamics geostatistical model that separates all regressive effects from the spatiotemporal interrelations. More precisely, the f-HDGM is defined by

1 $$\begin{aligned} y_{s,t}(h) = \mu _{s,t}(h) + \omega _{s,t}(h) + \varepsilon _{s,t}(h) , \end{aligned}$$

where the fixed effects component $\mu _{s,t}(h)$, the random effects component $\omega _{s,t}(h)$ and the modelling errors variance $\sigma ^2(h) = Var(\varepsilon _{s,t}(h))$ are modelled using splines.

**Fig. 1:**
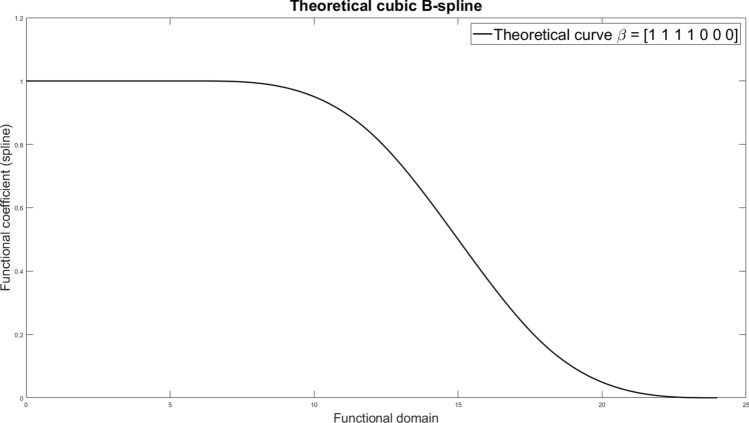
Cubic B-splines functional coefficient with seven basis functions and coefficients equal $\beta = (1, 1, 1, 1, 0, 0, 0)'$ used in the simulations

Let $B_{k,a}(h)$ be the *k*-th of the $K_a$ basis functions of component $a \in \{\mu , \omega , \sigma \}$. In Eq. 1, the mean, or the fixed effects component, is a linear regression model in the functional domain. That is,

2 $$\begin{aligned} \mu _{s,t}(h) = {\sum _{j = 0}^{p}}\sum _{k = 1}^{K_\mu } X_{s,t,j}(h) B_{k,\mu }(h) \beta _{jk} , \end{aligned}$$

where $X_{s,t,j}(h)$ denotes the functional observations of the *j*-th regressor, with $j=0$ referring to the functional intercept, and $j=1,\ldots ,p$ referring to the *p* functional covariates. For the generic *j*-th regressor, by multiplying of the spline basis matrix by the coefficients $\beta _j = (\beta _{j1},\ldots ,\beta _{jK_\mu })'$, we obtain the functional coefficients shown in Fig. 1. In Sect. 3, we propose an adaptive LASSO procedure to penalize these regression coefficients. In a nutshell, whether all entries of the vector $\beta _j$ are shrunk to zero or not, we can select the relevant regressors. That is if $\beta _j$ contains only zeros, then, the *j*-th regressor is removed from the model. Moreover, if $\beta _j$ is only partly shrunk to zero, we can select the relevant parts and knots of the *j*-th regressor in the functional domain.

In Eq. 1, the spatiotemporal dependence is modelled by the functional random effects $\omega _{s,t}(h)$, given by

3 $$\begin{aligned} \omega _{s,t}(h) = \sum _{k = 1}^{K_\omega } B_{k,\omega }(h) z_{s,t,k} . \end{aligned}$$

In Eq. 3, the latent component $z_{s,t} = (z_{s,t,1},\ldots ,z_{s,t,K_{\omega }})$ follows a temporal Markovian process, i.e.,

4 $$\begin{aligned} z_{s,t} = G z_{s,t-1} + \eta _{s,t} , \end{aligned}$$

where $\eta _{s,t}$ is a spatially correlated $K_\omega$-dimensional zero-mean Gaussian process

5 $$\begin{aligned} \eta _{s,t} \sim N_{K_\omega }(0, \Gamma ) . \end{aligned}$$

Let $\rho (d,\theta )$ be the exponential covariance function at distance *d* with spatial range $\theta$. Then, the spatial covariance function $\Gamma$ at location *s* and $s'$ is given by

6 $$\begin{aligned} \Gamma = \text {diag}(v_1\rho ({s}-{s}^\prime ; \theta _1), \ldots , v_{K_\omega }\rho ({s}-{s}^\prime ; \theta _{K_\omega })), \end{aligned}$$

where $v_i$ and $\theta _i$ are the variance and the range of the *j*-th component of $\eta _{s,t}$, repectively, with $i=1,\ldots ,K_\omega$.

Eventually, the model errors $\varepsilon _{s,t}$ are assumed to be independent and identically normally distributed across space and time, but the error variance may vary across the functional domain as follows:

7 $$\begin{aligned} \sigma ^2(h) = \sum _{k = 1}^{K_\sigma } B_{k,\sigma }(h) \sigma ^2_{k} . \end{aligned}$$

Let $\beta = (\beta _1,\ldots , \beta _p)'$ be the stacked vector of the spline coefficient vectors of the fixed effects model, let $\theta = (\theta _1,\ldots ,\theta _{K_\omega })'$ be the stacked vector of the spatial ranges, and let $v = (v_1,\ldots ,v_{K_\omega })'$ be the stacked vector of random effects variances. Also, let $\psi = \{G, V, \theta , v, \sigma ^2\}$ be the set of all coefficients of the random effects, including the error term. Moreover, let *H* denote the Hessian matrix of the model’s log-likelihood. The full set of parameters $\{\beta , \psi \}$ is estimated by maximising the log-likelihood using the EM algorithm. Let $\{\hat{\beta }_{MLE}, \hat{\psi }_{MLE}\}$ denote the maximum likelihood estimate of $\{\beta , \psi \}$. Moreover, let $H_{MLE} = H(\hat{\beta }_{MLE}, \hat{\psi }_{MLE})$ denote the Hessian matrix computed at the ML solutions $\{\hat{\beta }_{MLE}, \hat{\psi }_{MLE}\}$. The EM algorithm used for computation is implemented in the D-STEMv2 software (Finazzi and Fassò 2014; Wang et al. 2021) within the MATLAB environment. Starting with initial parameters $\{\beta ^{\langle 0 \rangle }, \psi ^{\langle 0 \rangle }\}$, the expectation of the complete data likelihood of the f-HDGM is iteratively maximised until the parameters converge or an upper bound of iterations is reached. Both, the convergence distance and the iteration limit, can be specified by the user. Moreover, the M-step involves the Kalman smoother estimating the random effects and possibly missing values. For details on the update steps, we refer the reader to Calculli et al. (2015) which is the basis of the algorithm implemented in D-STEMv2.

### Approximations

Estimating the parameters of the f-HDGM can be computationally demanding. Following Wang et al. (2021), we will consider two approximations in order to reduce the computational time. In the first approximation, the variance-covariance matrix of the parameters is computed using an approximated approach. This task is performed by fixing a threshold for the overall improvement in the variance-covariance matrix computation (see Sect. 2.5 of Wang et al. 2021).

The second approximation concerns a spatial partitioning approach. According to Stein (2013), we divide the complete dataset into *k* groups (based on the geodesic distance) of size *r*, and assume that the data in the different groups are not correlated. This implies a factorised likelihood function and the possibility of computing the E-step in parallel. As a result, the computational complexity is reduced from $O(Tn^3b^3)$ to $O(Tkr^3b^3)$ (see Sect. 2.4 of Wang et al. 2021). Moreover, if the computing infrastructure can handle *k* parallel processes, the computing time may be further reduced to $O(Tr^3b^3)$.

## Spatiotemporal adaptive LASSO estimation for functional coefficients

In this section, we suggest an adaptive LASSO approach to select (1) the relevant regressors, (2) the relevant sections of the functional coefficients and (3) the relevant knots of the fixed effects functional model $\mu _{s,t}(h)$. The emphasis is on modelling the relationship between the covariates and the response variable. Therefore, the parameters of the random effects components are kept unpenalised. Moreover, for regularised regression approaches, the covariance matrix of the model errors is usually not penalised (e.g., Fan and Li 2001; Tibshirani 1996).

Spatiotemporal parameters could also be included in the penalised procedure (e.g., see for random effects shrinking in linear mixed models Bondell et al. 2010). However, in this case, the shrinkage target should be adjusted to the specific empirical case. Indeed, while the temporal dependence parameter matrix *G* could be shrunk to zero, i.e., in the case of temporal independence, a zero shrinkage target is not meaningful for the variance parameters and the range parameter of the spatial dependence $\theta$.

We follow a penalised maximum likelihood estimation strategy for the fixed effects coefficients conditional on the random effects parameters, i.e.,

8 $$\begin{aligned} \hat{\beta }_{PMLE}(\lambda , \psi ) = \arg \max _{\beta } \mathcal {L}(\beta , \psi ) - \lambda f(\beta ) \, \end{aligned}$$

with the likelihood function $\mathcal {L}$ and a penalty function *f*. To reduce the computational burden, we locally approximate the full model log-likelihood $\mathcal {L}$ in (8) around the unpenalised and consistent ML estimates using a second-order local quadratic approximation (Jennrich and Sampson 1976; Longford 1987). That is, we obtain the approximated log-likelihood as follows:

9 $$\begin{aligned} \mathcal {L}(\beta ) \cong \mathcal {L}(\hat{\beta }_{MLE}) + \frac{1}{2} (\beta - \hat{\beta }_{MLE})' H_{MLE} (\beta - \hat{\beta }_{MLE}), \end{aligned}$$

where $$H_{MLE} = \nabla ^2 \mathcal {L}(\hat{\beta }_{MLE}, \hat{\psi }_{MLE})$$.

Similar computational solutions involving local approximation of the likelihood have been proposed by Zou and Li (2008) for obtaining penalised estimates of the parameters in Generalised Linear Models (GLM) via the one-step sparse estimator by Fan and Li (2001) for variable selection adopting nonconcave penalties; by McIlhagga (2016) for penalized GLM based on Fisher scoring algorithms; by Zhu et al. (2010) for adaptive spatial LASSO in lattice data; and by Reyes et al. (2012) for penalised likelihood problems in linear spatiotemporal contexts. This study extends the aforementioned studies by obtaining penalised estimates of the fixed effects coefficients of a linear mixed model for functional data, with the spatiotemporal dynamics modelled by a geostatistical random component.

We now consider the penalty function $f(\beta )$ in Equation (8). Motivated by the oracle properties of the adaptive LASSO estimates (Zou 2006; Bondell et al. 2010), we use an adaptive penalty for the likelihood of the functional HDGM. Because the observed data are supposed to be correlated in space and time, it is important that the algorithm be selection-consistent (Zhang 2010) even in the case of correlated observations. However, this may not often be the case for classic LASSO approaches (see among others, for conditions of selection consistency Zhao and Yu 2006). Thus, we suggest an adaptive LASSO penalty that has the desired property of selection consistency, as shown by Zou (2006); Zou and Li (2008); Huang et al. (2008).

We propose the following estimator with an adaptive LASSO penalty for the fixed effects coefficients of the f-HDGM:

10 $$\begin{aligned} \hat{\beta }_{PMLE}(\lambda ; \hat{\psi }_{MLE})= & {} \arg \min _{\beta } - \frac{1}{2} (\beta - \hat{\beta }_{MLE})' H_{MLE} (\beta - \hat{\beta }_{MLE}) \nonumber \\{} & {} + N \lambda \vert \vert w \, \circ \, \beta \vert \vert _1 \quad \, \end{aligned}$$

where $\lambda$ is the regularisation parameter, $\circ$ is the element-wise product, and the penalty weights *w* are chosen as the inverse initial ML estimates, that is, $w = (w_i)_{i=1,\ldots ,p}$ with $w_i = \frac{1}{\vert \hat{\beta }_{MLE, i}\vert ^{\gamma }}$, with $\gamma = 1$ and $\hat{\beta }_{MLE, i}$ being the *i*-th entry of $\hat{\beta }_{MLE}$. To increase or diminish the influence of the initial estimates, $\gamma \ge 0$ could also be chosen differently. Generally, to obtain the oracle properties, the penalty parameter $\lambda$ should be of order $\sqrt{n}$ (see Zou and Li 2008). In the next paragraph, we will go into further details on the selection of $\lambda$.

The algorithm used to solve minimisation in (10) is based on the BFGS quasi-Newton method over the non-zero coefficients, that is, the so called active set, with the initial values being $\hat{\beta }_{MLE}$. The algorithm requires limited computation effort, as the time consuming computation of the Hessian matrix $H_{MLE}$ used in the approximation (9) is done only once, and the second-order derivatives can be computed numerically throughout the optimisation. Notice that the dimension of problem (10) is much smaller compared to the full model MLE as it involves only *beta*, while $\psi$ is kept fixed at $\hat{\psi }_{MLE}$.

The penalised procedure shrinks irrelevant coefficients to zero. Because this applies to all basis functions separately, we do not impose that all coefficients associated with one regressor must be shrunk to zero simultaneously as for a block LASSO approach. It is then possible to select the relevant sections of a functional coefficient and exclude the irrelevant knots. However, the basis functions may overlap to some extent. This implies that the height of the functional coefficient at a given point in the functional domain (i.e., the sum of the weighted basis functions at a given point) is determined by several coefficients. If only some of such coefficients are zero, the functional coefficient is not zero. Hence, typically, smooth transitions shrunken to zero can be observed in the functional domain, depending on the number and location of the knots (i.e., the fewer knots there are, the smoother the estimated function is). This further encourages the use of an adaptive LASSO penalty, which leads to asymptotically unbiased estimates (see Zou 2006).

### Cross-validation

The penalty parameter $\lambda$ is determined by minimising a prediction error metric, say PE, obtained from a random K-fold cross-validation (CV) study. For this reason, let $\mathcal {D} = \{y_{s,t}(h), s \in S, t=1,\ldots ,T\}$ be the set of all available functional observations, and let $\mathcal {D}_1,\ldots ,\mathcal {D}_K$ be a random partition of $\mathcal {D}$, which is made by randomly assigning *N*/*k* observation to each group $\mathcal {D}_i$ with $i=1,\ldots ,K$.

For each subset $\mathcal {D}_i$, the penalised estimation is performed for a certain predefined sequence of $\lambda$, including $\lambda = 0$. In particular, the parameters are estimated according to (10) using data in $\mathcal {D}-\mathcal {D}_i = \{s \in \mathcal {D}: s \notin \mathcal {D}_i \}$. Then, the data in $\mathcal {D}_i$, are used to evaluate the out-of-sample prediction $\hat{y}_{s,t}(h,\lambda )$ for a fixed $\lambda$, and the corresponding prediction error metric PE$(\lambda )_i$. Eventually, for each $\lambda$, the overall performance measures $PE(\lambda )$ is obtained by averaging $PE(\lambda )_i$ for $i=1,2,\ldots ,K$. The optimal $\lambda ^*$ may be chosen either by minimising PE, namely

$$\begin{aligned} \lambda ^*_{min,PE} = argmin_\lambda (PE(\lambda )) \end{aligned}$$

In order to consider the random nature of sets $\mathcal {D}_i$, a popular solution is to adopt the so-called one-standard-error rule, namely to chose $\lambda$ defined by

$$\begin{aligned} \lambda ^*_{1\sigma ,PE} = inf_\lambda \{PE(\lambda ) \ge PE(\lambda ^*)+\sigma (PE)\} \, \end{aligned}$$

where $\sigma (PE)$ is the standard error of the average of the $\hbox {PE}_i$. Of course any $\lambda ^*$ between the above two values would have the same statistical validity. The choice of the metric PE may be based on the application at hand. Popular choices are the root mean square error and the mean absolute error which are discussed in the following Sect. 3.2.

For a fixed choice of PE and criterion for $\lambda ^*$, the procedure is synthesised using the pseudo-code in Algorithm 1.
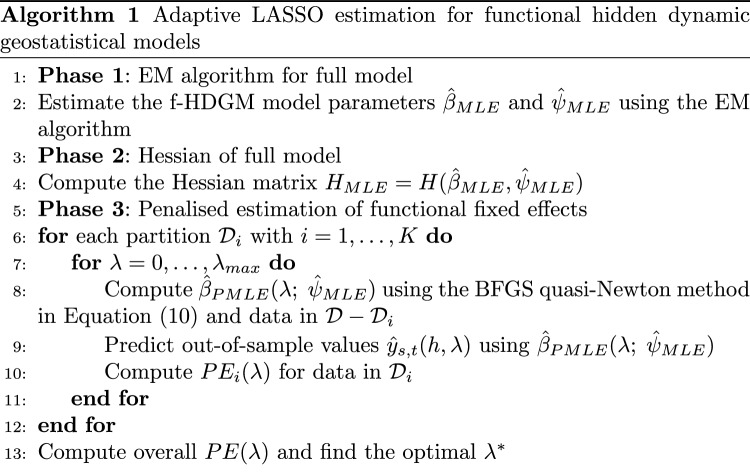


### Which prediction error metric?

In the simulations and application of the next sections, we will use the root-mean-square error (RMSE) and the mean absolute error (MAE) as prediction error metrics PE. For each random partition $\mathcal {D}_i$, they are defined as follows:

11 $$\begin{aligned} RMSE(\lambda )_i= & {} \frac{1}{24\Vert \mathcal {D}_i\Vert } \sum _{y_{s,t} \in \mathcal {D}_i}\sum _{h \in H}{(y_{s,t}(h)-\hat{y}_{s,t}(h,\lambda ))^2} \end{aligned}$$

12 $$\begin{aligned} MAE(\lambda )_i= & {} \frac{1}{24\Vert \mathcal {D}_i\Vert } \sum _{y_{s,t} \in \mathcal {D}_i}\sum _{h \in H}{ |y_{s,t}(h)-\hat{y}_{s,t}(h,\lambda ) |} \end{aligned}$$

where $\hat{y}_{s,t}(h,\lambda )$ is the prediction provided by the estimated model for a fixed $\lambda$, and $\Vert \mathcal {D}-\mathcal {D}_i\Vert$ denotes the cardinality of a the set $\mathcal {D}_i$. Although other alternatives are available (Chicco et al. 2021), RMSE and MAE are usual metrics used in the literature (Sammut and Webb 2010b, a).

In this paper, we employ both RMSE and MAE without stating a preference rule between the two as there is no universal consensus on the most appropriate metric for model errors (Cort and Kenji 2005). The simulation study described in Sect. 4 is built under the Gaussianity assumption of Sect. 2. Instead, the air quality exercise of Sect. 5 does not fulfill such assumption. As discussed in Chai and Draxler (2014), the RMSE is appropriate when the error is Gaussian distributed. Whereas, when the error distribution is affected by skewness, outliers or is leptokurtic (Karunasingha 2022), MAE is preferred being more robust (Willmott et al. 2009; Hodson 2022). Therefore, we develop the analysis using both metrics as suggested by Chai et al. (2009).

Using $\lambda ^*_{min,PE}$ provides the better predictive capabilities for the data at hand. Instead, the one-standard-error rule leads to a parsimonious set of parameters and the selected model is simpler and more interpretable than previous one (Hastie et al. 2017, 2015). Therefore, in both simulations and the application we will compare the results coming from the four CV criteria: $\lambda ^*_{min,RMSE}$, $\lambda ^*_{min,MAE}$, $\lambda ^*_{1\sigma ,RMSE}$, and $\lambda ^*_{1\sigma ,MAE}$.

## Monte carlo simulation study

In this section, we present and discuss an extensive Monte Carlo simulation study aimed at evaluating the performance of the algorithm in various contexts with increasing levels of complexity and representative of common application contexts.

### Experimental design

To evaluate the performance of the model selection algorithm, we perform a Monte Carlo simulation study based on three settings, labelled as *Setting I*, *Setting II*, and *Setting III*. The three schemes are summarised in Table 1. The settings represent the following situations of interest in geostatistical models. First, we regard a multiple regression model as a benchmark approach (i.e., all temporal and spatial dependence parameters are chosen such that the resulting process is independent in space and time). Second, we consider the case of a response variable that is correlated across space and time but with uncorrelated regressors. Third, in *Setting III*, we introduce cross-correlation among the regressors. In particular, we consider cross-correlation ranging from moderate (0.5) to strong (0.9). The latter setting represents the most challenging for model selection, but also the most realistic one.

The spatial dependence is exponentially decreasing with spatial range $\theta = 50$ km, implying a correlation is below 0.37 beyond a 50 km distance. The temporal autoregressive coefficients in the *G* matrix are all equal to 0.85, resulting in a pronounced temporal persistence, which is common in meteorological-related applications.

To represent a realistic spatial setting, we take the coordinates from the data that is used in the following empirical sections. More precisely, the coordinates of the spatial locations refer to the atmospheric monitoring sites belonging to the 84-stations network of *ARPA Lombardia* (see the paper by Maranzano 2022). Regarding the temporal resolution, we consider that the data are observed over 365 days, with each day representing the functional domain.

For each of the three settings, 500 Monte Carlo replications are simulated using a random subset of 15 locations extracted from the full list of ARPA Lombardia monitoring network.

**Table 1:** Specification of the simulation settings

	Setting I	Setting II	Setting III
Description	Uncorrelated response	Spatiotemporal correlation	Spatiotemporal correlation
	Uncorrelated predictors	Uncorrelated predictors	Correlated predictors
Spatial locations *s*	15	15	15
Days *t*	365	365	365
Functional domain	[0, 24]	[0, 24]	[0, 24]
Total observations	$15 \cdot 365\cdot 24$	$15 \cdot 365\cdot 24$	$15 \cdot 365\cdot 24$
Covariates	${\textbf {X}} \sim N_3({\textbf {0}},\Sigma _X)$	${\textbf {X}} \sim N_3({\textbf {0}},\Sigma _X)$	${\textbf {X}} \sim N_3({\textbf {0}},\Sigma _X)$
Var-cov matrix	$$\Sigma _X = \left[ \begin{array}{ccc} 1 &{} 0 &{} 0 \\ 0 &{} 1 &{} 0 \\ 0 &{} 0 &{} 1 \end{array} \right]$$	$$\Sigma _X = \left[ \begin{array}{ccc} 1 &{} 0 &{} 0 \\ 0 &{} 1 &{} 0 \\ 0 &{} 0 &{} 1 \end{array} \right]$$	$$\Sigma _X = \left[ \begin{array}{ccc} 1 &{} 0.9 &{} 0.7 \\ 0.9 &{} 1 &{} 0.5 \\ 0.7 &{} 0.5 &{} 1 \end{array} \right]$$
Spline type	B-spline	B-spline	B-spline
Interior knots	5	5	5
Spline order	3 (cubic)	3 (cubic)	3 (cubic)
Number of bases	7	7	7
$\beta$ coefficients	[1 1 1 1 0 0 0]	[1 1 1 1 0 0 0]	[1 1 1 1 0 0 0]
$\theta$	0 km	50 km	50 km
**G**	diag(0,0,0)	diag(0.85,0.85,0.85)	diag(0.85,0.85,0.85)
$\Sigma _\eta$	diag(0,0,0)	diag(1,1,1)	diag(1,1,1)
$\Sigma _\epsilon$	diag(1,1,1)	diag(1,1,1)	diag(1,1,1)

For the functional interpolation, we use a simple set-up of cubic B-spline basis functions (Ramsay 2005) with 7 knots, corresponding to the $\beta$ coefficients given by $\beta =(1 1 1 1 0 0 0)$. This allows us to analyse the performance of the algorithm in selecting relevant parts across the functional domain, we considered functional regression coefficients, that is 1 at the start of a day and going smoothly to 0, as shown in Fig. 1. Note that B-splines used do not reproduce daily periodicity. This will be addressed using Fourier bases in the application.

The simulated values of the response variable are given by the sum of the random effect, the measurement error, the linear combination of the three covariates, and the functional intercept. Tibshirani (1996) suggest to standardise all the covariates and centering the dependent variable before applying the penalised regression. In our simulation design, all the covariates are simulated by a Gaussian distribution with zero mean and unit variance, thus no standardisation is needed. Since we are interested in the daily profile, we keep the response variable in its original scale and the penalty is only applied to the three covariates, while the functional intercept is left unpenalised. In this way, even for high penalty values that set the spline coefficients to zero, the intercept can still capture the average intraday pattern.

The penalty term sequence was generated according to an exponentially decaying grid, starting from $\lambda _{min} = 10^{-5}$ up to $\lambda _{max} = 0.5$. As we will show in the simulation results, for a value of $\lambda$ greater than 0.5, all the coefficients shrunk to 0. We added as the first value of the sequence $\lambda = 0$, corresponding to the unpenalised maximum likelihood solution. In total, we consider 101 different values. To identify the optimal value of $\lambda$, we perform a 10-fold random cross-validation across space and time.

Being a simulation experiment, we are able to determine the range of possible values in which the prediction error metrics used (i.e., RMSE and MAE) may occur. Based on the simulation setup described in Table 1, we simulate reference values for the minimum and maximum of each prediction error metric in each setting. Reference values are reported in Table 2. The reported reference values correspond to the average value of the minima and maxima of the three prediction error metrics calculated on $n=100$ simulations of each of the three settings. As may be noticed, both the spatiotemporal dependence and the cross-correlation among the covariates increase both the minimum and the maximum. In addition to the mean value, under parenthesis we report the simulation standard errors.

**Table 2:** Simulated ($n=100$ simulations) reference values for each setting. Reported values are the average across simulations of the minimum (*min*) and maximum (*max*) MSE, RMSE, and MAE. Values under parenthesis are the standard error of the mean computed across simulations

		MSE	RMSE	MAE
Setting I	min	0.9999	1.0000	0.7979
		(0.0004)	(0.0002)	(0.0002)
	max	2.7257	1.6510	1.2724
		(0.0013)	(0.0004)	(0.0003)
Setting II	min	0.9989	0.9995	0.7975
		(0.0004)	(0.0002)	(0.0002)
	max	2.7238	1.6504	1.2719
		(0.0013)	(0.0004)	(0.0003)
Setting III	min	0.9996	0.9998	(0.7977
		(0.0004)	(0.0002)	(0.0002)
	max	5.1389	2.2669	1.6956
		(0.0022)	(0.0005)	(0.0003)

### Simulation results

**Fig. 2:**
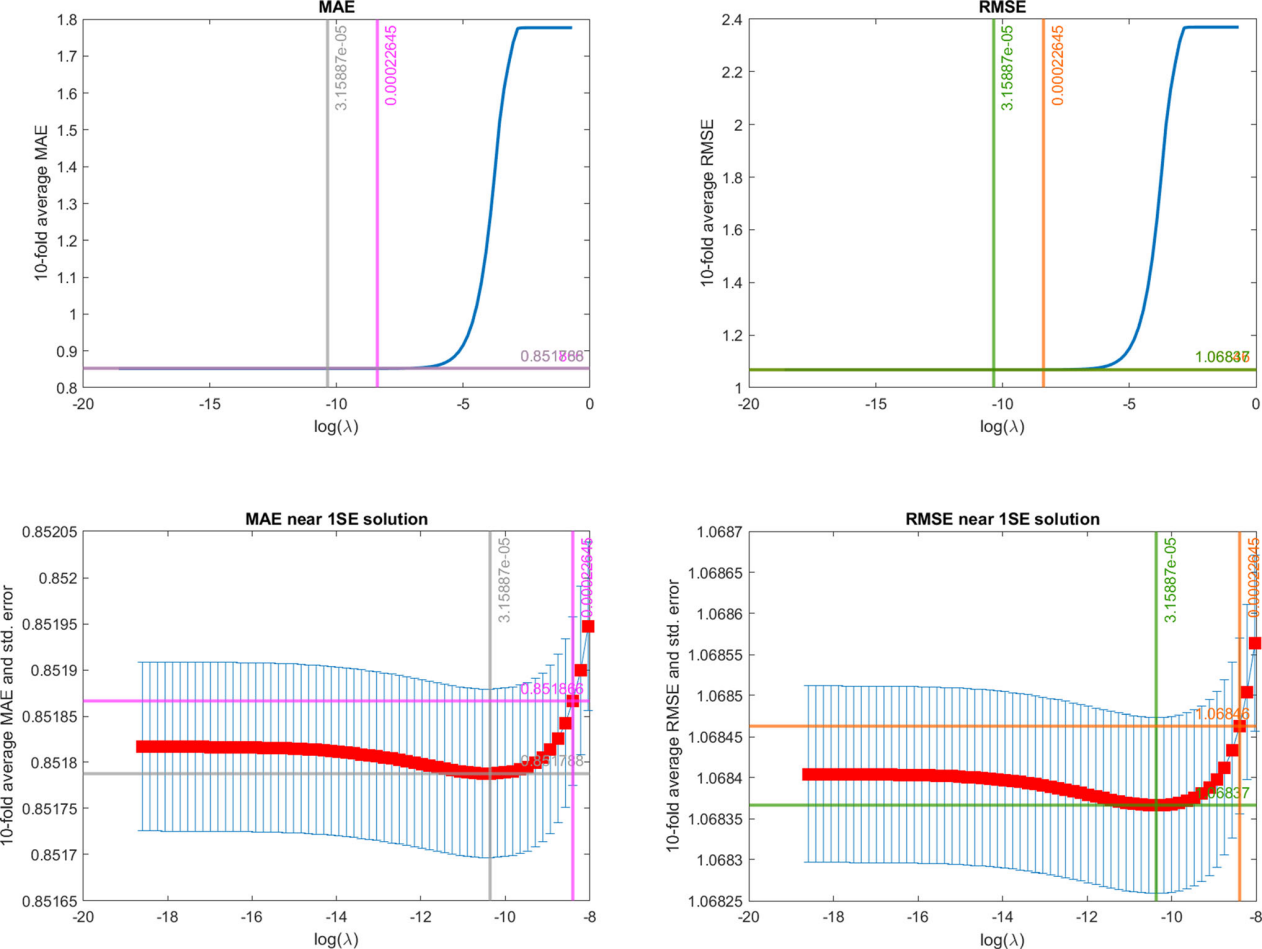
RMSE and MAE for different values of $\lambda$ in Setting III. Top panels: full $\lambda$ range. Bottom panels: near-optimum $\lambda$ range. Left panels: MAE. Right panels: RMSE. The vertical and horizontal lines correspond to the considered selection rules (grey: $\lambda ^*_{1min,MAE}$; pink: $\lambda ^*_{1\sigma ,MAE}$; black: $\lambda ^*_{min,RMSE}$; orange: $\lambda ^*_{1\sigma ,RMSE}$)

The common features of the three settings are numerous. First, we observe that both the presence of spatiotemporal dependence (Settings II and III) and cross-correlation between covariates (Setting III) increase the values of RMSE and MAE. This is reflected in the RMSE and MAE values estimated by penalisation (Fig. 2), whose minimum and maximum values are perfectly overlapping with the reference values (Table 2) for Setting I. However, not surprisingly, the estimated values of the metrics in Setting II and Setting III are slightly higher than the reference values. In fact, it is known that when spatiotemporal dependence occurs, part of the information is duplicated or redundant, thus the information embedded in the sample is lower compated to the case of independent data (Lee and Lund 2008; Griffith 2005). This results in an increase of the prediction errors variability (i.e., the non-predictable portion of the data).^1^ Also, as expected, for all three settings, the penalised optimal estimates ($\lambda ^*_{min,RMSE}$ and $\lambda ^*_{min,MAE}$) are always different from the maximum likelihood estimates and are mutually consistent (i.e., in line with the Gaussian framework, the $\lambda$ value minimising the two metrics is on average the same). However, from the purely predictive capability standpoint, penalised and unpenalised are equivalent (i.e., the confidence intervals for $\lambda =0$, $\lambda ^*_{min,RMSE}$ and $\lambda ^*_{min,MAE}$ overlap). Last but not least, the one-standard-error metrics lead to coefficient estimates that are much closer to the true values compated to the MLE and minimum solutions.

Specifically for Setting III, the major insights are summarised as follows. Figure 2 shows the average RMSE and MAE across the Monte Carlo replications and the optimal $\lambda$ values obtained using the four above mentioned criteria. The upper panels show MAEs (left) and RMSEs (right) for all the whole range of $\lambda$ values considered, while the lower panels focus on the behaviour of MAE and RMSE near the optimum solutions. Moreover, we depict the CV variability with the error bars, computed as plus and minus one standard error of the CV prediction error. Both RMSE and MAE plots clearly show that $\lambda$ minimising RMSE or MAE provides different performances from the MLE solution. Overall, both the RMSE and MAE show smooth patterns. Consistently with the Gaussian simulation framework, the $\lambda$ minimising the metrics (grey and green vertical lines) coincide, as well as the penalties associated with the one-standard-error rule (pink and orange vertical lines). As previously stated, in terms of predictive capacity, each model with $\lambda$ lying between the MLE and the one-standard-error solutions are equivalent as the corresponding intervals overlap.

**Fig. 3:**
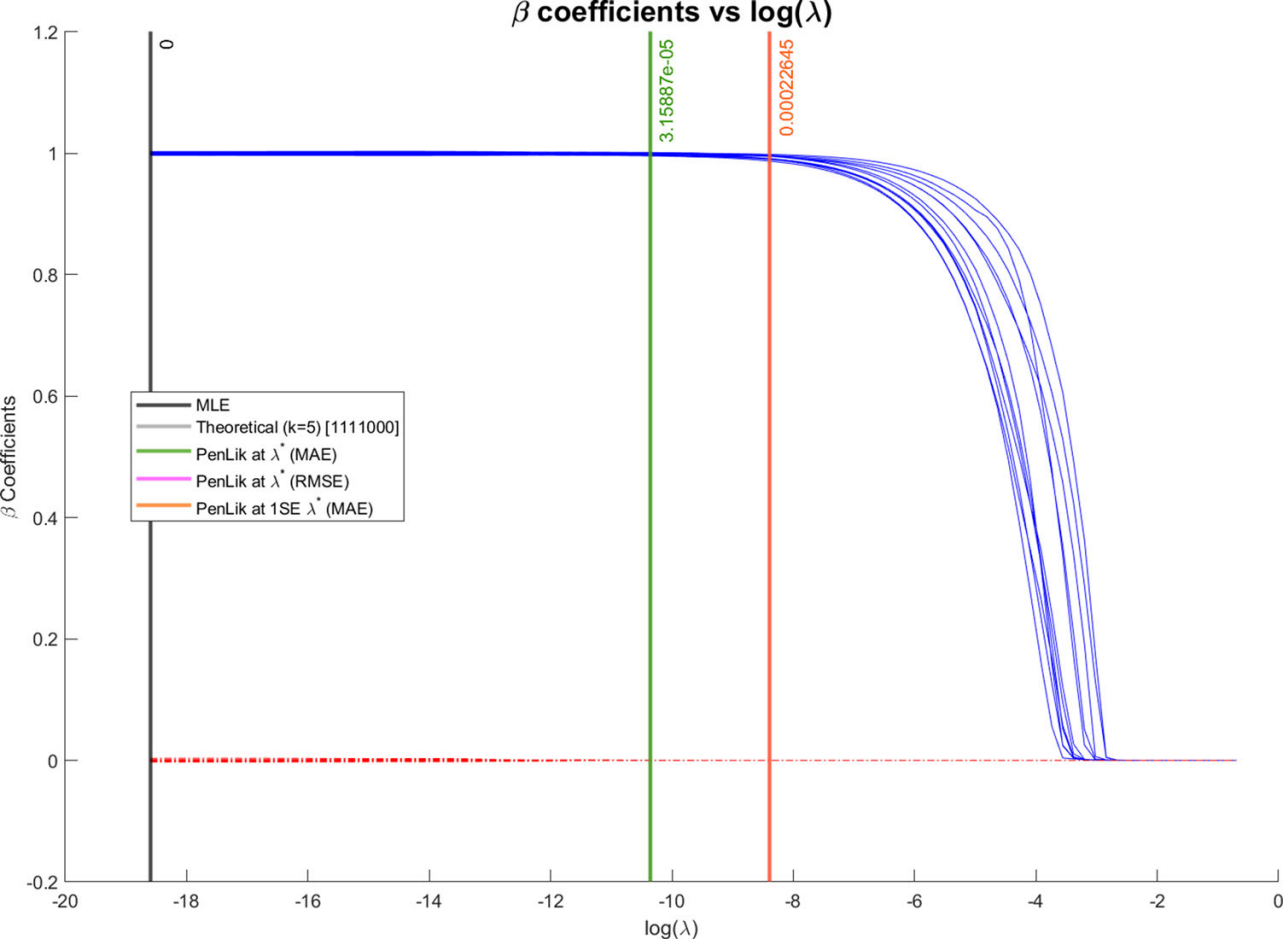
Average estimated coefficients for different values of $\lambda$ in Setting III. The positive coefficients are drawn in blue, and the zero coefficients are depicted by the red dashed lines

In Fig. 3 we can observe that the coefficients averages are smoothly shrunk towards 0. For values of the penalty term $\lambda$ greater than 0.03 (i.e., $log(\lambda ) > -3.50$), all the coefficients are shrunk to 0 and the RMSE and MAE stabilise around 2.35 and 1.75, respectively.

**Fig. 4:**
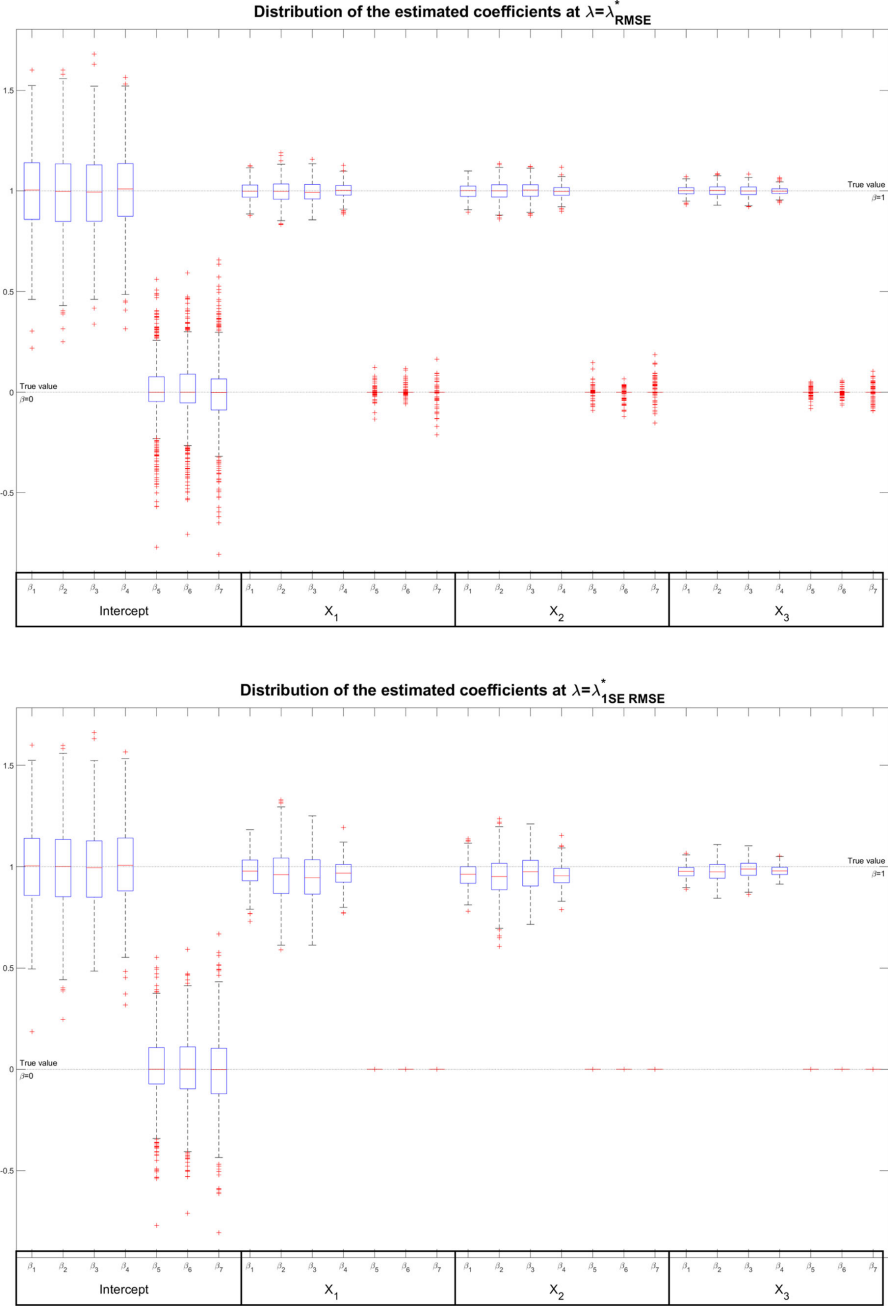
Box plot of the estimated coefficients across 500 simulations at $\lambda ^*_{min,RMSE}$ (upper panel) and at $\lambda ^*_{1\sigma ,RMSE}$ (lower panel) for Setting III

In Fig. 4, we plot the empirical distribution (i.e., the box-plot) across the simulations of each fixed effects coefficient for $\lambda = \lambda ^*_{min,RMSE}$ (upper panel) and $\lambda = \lambda ^*_{1\sigma ,RMSE}$ (lower panel).

In both cases, the following is very noticeable: (1) the coefficients are estimated very close to their actual value, which indicates that the penalised estimators are approximately unbiased; (2) the variability for null coefficients is considerably smaller than for coefficients equal to 1; and (3) for increasing values of penalisation (i.e. moving from $\lambda ^*_{min,RMSE}$ to $\lambda ^*_{1\sigma ,RMSE}$ solutions) the coefficients variability decreases, especially for the true zero coefficients. The two last considerations are strengthened by Table S5 in the Supplementary Information. The findings suggest that the one-standard-error estimates (for both MAE and RMSE) are quite different from the minimum and MLE solutions. In fact, it is easy to see that the coefficients associated with bases 5, 6 and 7 of each covariate (all null in the simulative setup) tend to the true value 0 as the penalty increases, while the first four coefficients remain around the true value 1. In the case of the null parameters, comparing the distances between the estimators and the true coefficients ("RMSE" columns), it is noticeable tht the distance decreases as one moves from the MLE estimators to the penalized ones (reaching exactly zero with the standard-error estimators). In contrast, high penalty values introduce a bias in the one-unit coefficients, leading to an increase in their variability. Thus, we can state that the one-standard-error rule enjoy an oracle property in the sense that for the null coefficients we detect the true zero values. Also, from an inferential standpoint, the sparsity induced by the one-standard-error estimates leads to models less complex and easier to interpret than the MLE estimates.

## Application to air quality in Lombardy

In this section, we present an empirical application of the penalisation algorithm to an air quality assessment case study. The application is structured in such a way to study some scalability properties of the procedure, that is the computation time of the phases composing Algorithm 1, and its behaviour as the model complexity increases.

**Fig. 5:**
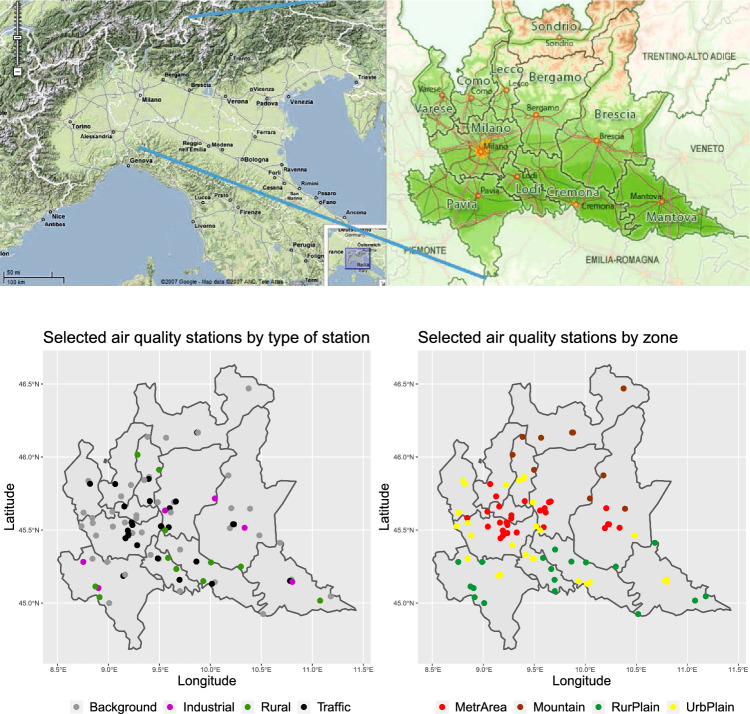
Physical map of Po Valley (upper left panel) and Lombardy (upper right panel) and the ARPA Lombardia air quality monitoring network by type of station (lower left panel) and type of area (lower right panel)

The application refers to air quality data recorded during the COVID-19 pandemic in Lombardy region, Italy (see the upper panel of Fig. 5). Airborne pollution in Lombardy has attracted considerable research interest for many years. With the COVID-19 emergence, many researchers have become increasingly interested in the short-term effects of lockdowns on air quality (Cameletti 2020; Collivignarelli et al. 2020; Lovarelli et al. 2020; Fassò et al. 2022).

**Fig. 6:**
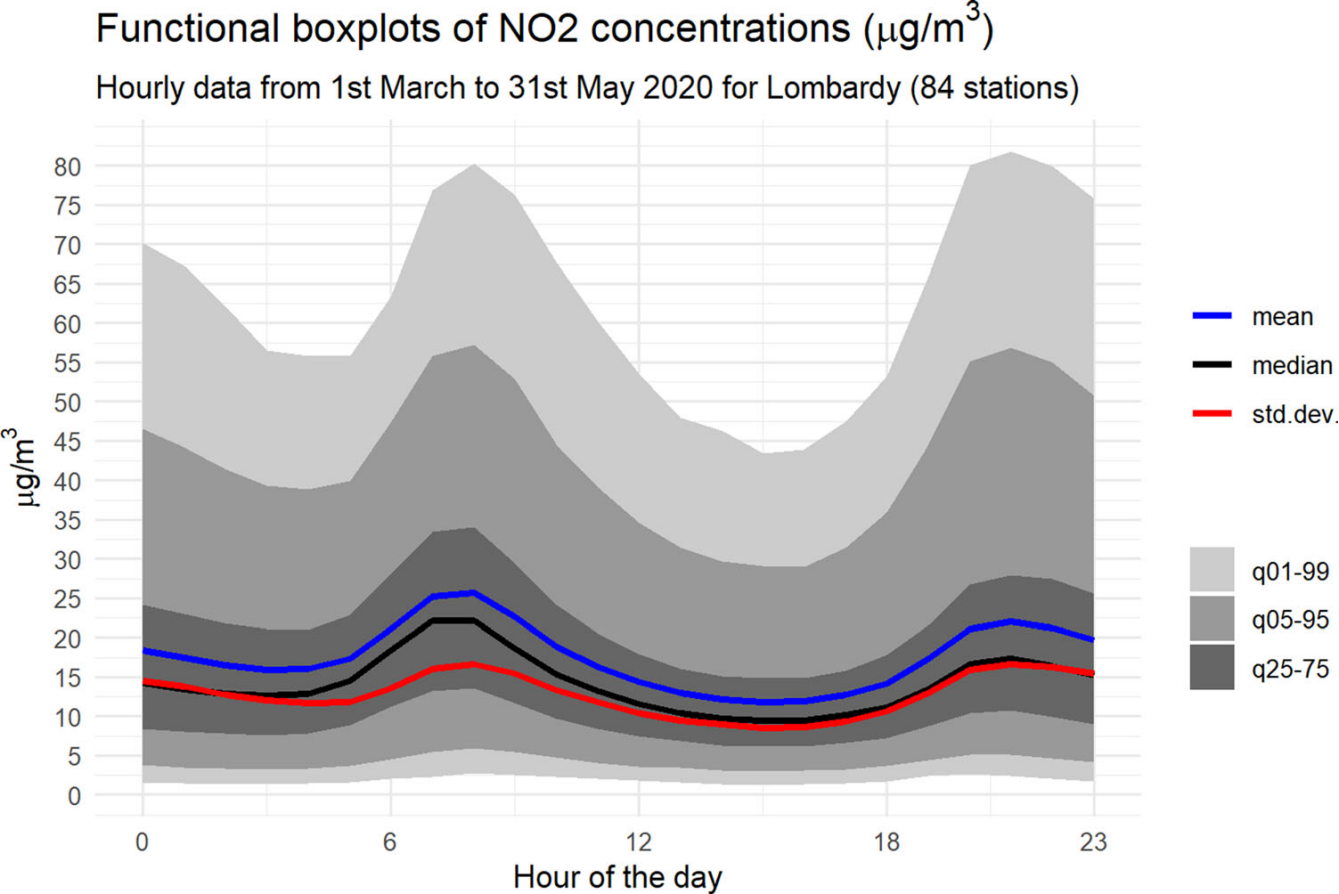
Intraday box-plot of $\hbox {NO}_2$ concentrations ($\mu g/m^3$) observed between the 1 March 2020 and 31 May 2020

We model hourly nitrogen dioxide ($\hbox {NO}_2$) concentrations obtained by the $n=84$ stations depicted in Fig. 5 (bottom panel) from 1 March, 2020, to 31 May, 2020, that is $T=92$ days. We consider the concentration throughout the day as a functional observation. For a first insight on the intraday variations, in Fig. 6 we show the regional intra-daily evolution of the $\hbox {NO}_2$ concentrations through a functional box plot computed on the full sample. The 24-hour profile clearly shows the intra-day dynamics of the average (blue curve) and median (black) concentrations, as well as of their variability (the red line represents the functional standard deviation). In particular, there are strong differences between the $\hbox {NO}_2$ concentrations at night and day. They are in accordance with anthropic activities – that is, very high concentrations are seen during peak hours, between 7am and 9am and between 5pm and 11pm. Also, the standard deviation’s curve shows a pattern very close to the average, with two local maxima at peak hours and the minimum value during the afternoon. On average, the standard deviation is bounded between 8.50$\mu g/m^3$ (3pm) and 16.62$\mu g/m^3$ (9pm), with mean 12.76$\mu g/m^3$.

To explain the airborne pollutant concentrations, we consider a set of nine meteorological and land cover variables: temperature ($^{\circ } C$), precipitation (mm), relative humidity ($\%$), atmospheric pressure (Pa), eastward and northward component of the wind (m/s), geopotential height ($\hbox {m}^2$/$\hbox {s}^2$) and high and low vegetation covering (measured as one-half of the total green leaf area per unit horizontal ground surface area, cf. Sabater 2019). Since the variables present different scales and ranges, we standardise both the response variable and the covariates with respect to their overall 24-hour mean and standard deviation. Following Tibshirani (1996), having standardised both the response variable and the covariates, penalisation is applied to predictors only, while the functional intercept is left unpenalised. The total number of observations was $n \times T = 185472$ for each variable.

To account for the natural daily cycle, in Equation (1), we set *t* as the day, whereas *h* is the time across the day. Hence, periodic Fourier basis functions with *b* bases and a support between 0 and 24 were used.^2^ Recall that by construction, Fourier splines require an odd number of bases, and their interpretation depends on the frequency. In fact, except for the first basis, the other basis pairs were calculated at increasing frequency. For example, if the number of bases was $b=5$, the pair formed by the fourth and fifth bases would have twice the frequency of the second and third pair. The use of Fourier bases ensures the continuity of the last hour of a day to the first hour of the consecutive day. According to the number of basis functions, the total number of parameters to estimate is equal to $b \times 14$. In particular, $b \times 10$ parameters are associated with the covariates and the functional intercept; $b \times 3$ with spatiotemporal dynamics, and *b* with residual component variances.

The algorithm is initialised by estimating both the fixed and random effects of the f-HDGM using the unpenalised MLE. After estimating the full model, we apply the penalised likelihood model selection algorithm using an exponentially decaying grid of penalty coefficients $\lambda$ that ranged from $\lambda _{min}=10^{-4}$ to $\lambda _{max}=0.50$. We also includes a value of $\lambda =0$ for the unpenalised estimates.

### Scalability of the algorithm

Using the air quality data introduced above, we study the computing time of Phases 1, 2 and 3 of Algorithm 1 and the algorithm’s behaviour for increasing model complexity. To do this, we consider an increasing number of Fourier bases *b* for each covariate and an increasing number of spatial partitions. We also consider the impact of approximation methods for the fixed-effect coefficients (i.e., $\hat{\beta }_{MLE}$ and the Hessian matrix (i.e., $H_{MLE}$) of the initial unpenalised f-HDGM introduced in Sect. 2.2.

Moreover, we examine the algorithm’s ability to select only the relevant frequencies of the Fourier bases by shrinking irrelevant frequencies towards 0. Thus, we test the following occurrences as the complexity increases: (1) the increase in the number of fixed zero coefficients, and (2) the higher concentration of zeroes for the coefficients associated with high Fourier frequencies. The latter is consistent with observed $\hbox {NO}_2$ concentrations (Fig. 6), whose intra-day behaviour is fairly smooth and shows two peaks, which mean that from a modelling perspective, a small number of seasonal frequencies (low complexity) is expected.

A total of 17 models were evaluated. For each model, we considered the computation time for the three main phases of the algorithm – that is, the initial model estimation with the EM algorithm, the computation of the variance-covariance matrix of the parameters, and the penalised likelihood algorithm.

For model complexity we consider three scenarios: $b=5$, $b=7$ and $b=9$. In the first case, the total number of the model’s parameters is $b \times 14 =$70; in the second case, is 98, and in the third case is 126. Therefore, the numbers of fixed effect parameters is 50, 70, and 90, respectively. Moreover, for spatial partitioning we consider groups varying from $k=1$ (no spatial partitioning) to $k=5$. Eventually, the threshold for the overall improvement in the variance-covariance matrix computation is fixed to 0.01.

**Fig. 7:**
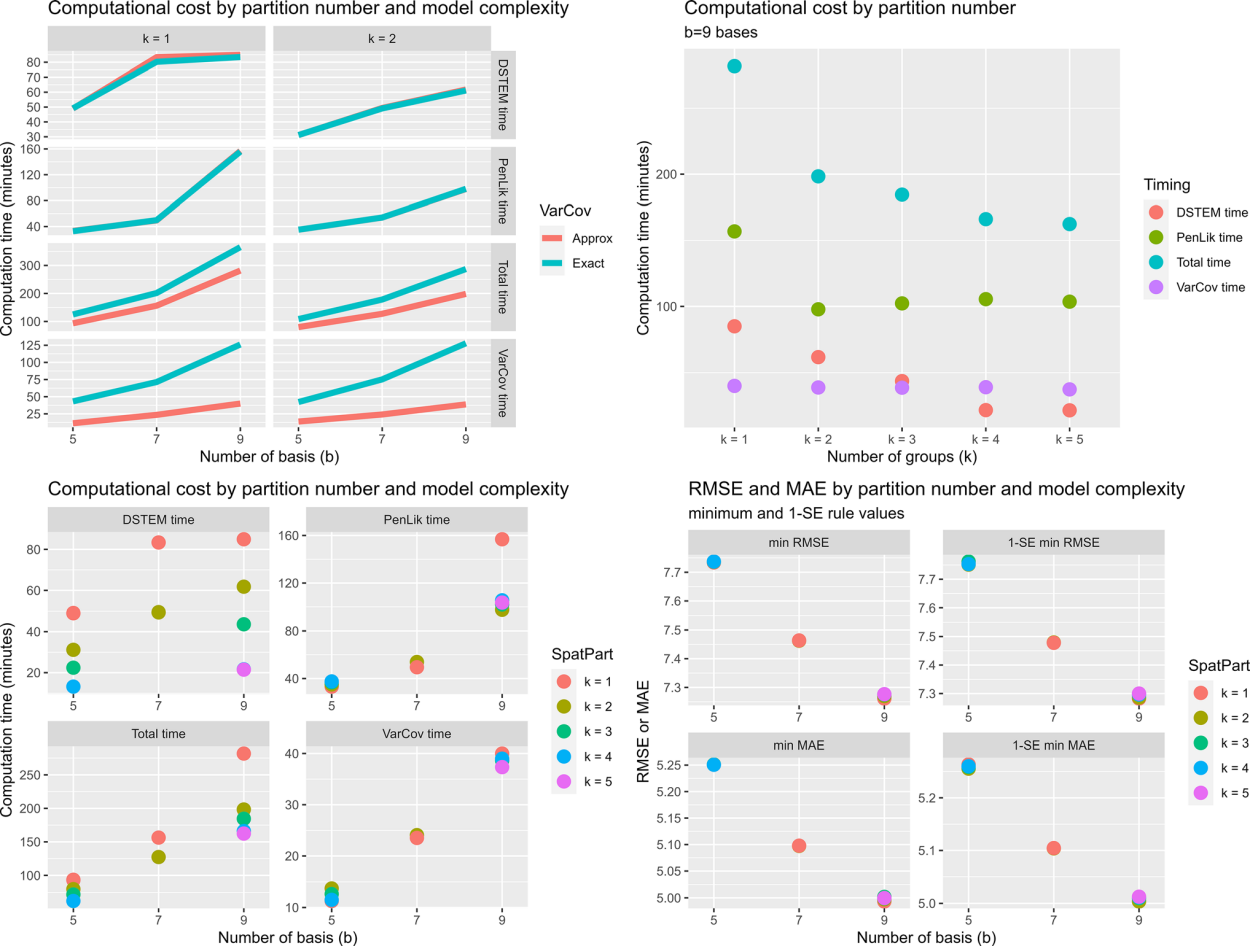
Computation time and cross-validation errors across the models. Computation time of each phase by model complexity with and without spatial partitioning (upper left panel); computation time of each phase by increasing level of spatial partitioning (upper right panel); computation time of each phase by increasing level of spatial partitioning and model complexity (lower left panel); RMSE and MAE by model complexity and increasing spatial partitioning (lower right panel)

**Fig. 8:**
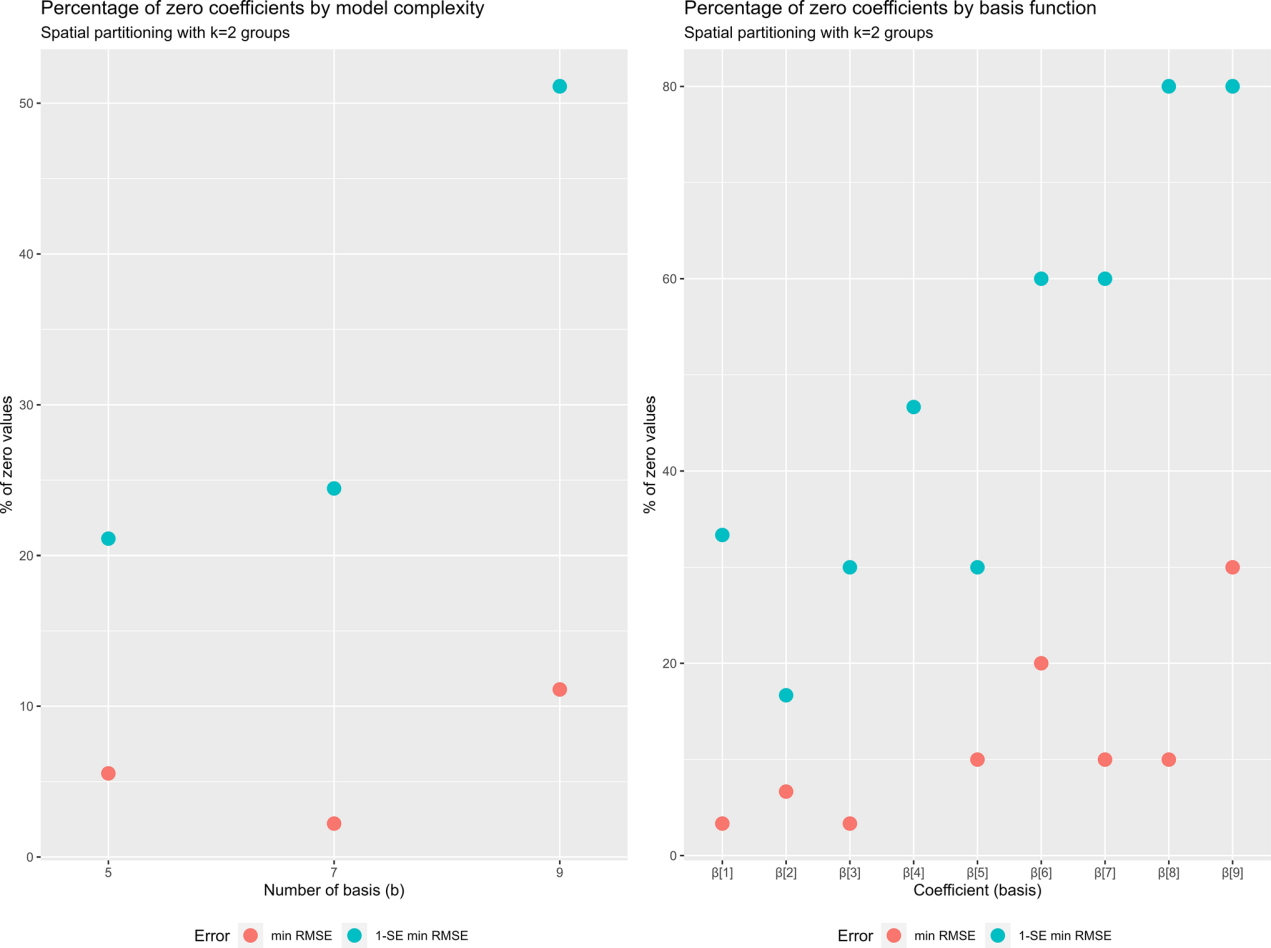
Percentage of zero coefficients across the models. Percentage of zero coefficients by model complexity when applying a spatial partitioning with $k=2$ groups (left panel); percentage of zero coefficients by basis function (coefficients) when applying a spatial partitioning with $k=2$ groups

The results are summarised in Figs. 7 and 8, and full details are given in Table S1 of the Supplementary Information.

In particular, Fig. 7 shows the computational cost of the penalised likelihood algorithm as a function of the model complexity, the spatial partitioning and the adoption of an approximation for the variance-covariance matrix. The main results are summarised as follows:

- Variance-covariance matrix approximation (upper left panel of Fig. 7): compared to the exact solution, the approximation reduces the computation time of 66% for Phase 2, and 25% for the overall computation. This holds independently from the model complexity. Of course, the penalisation algorithm is not affected;
- Spatial partitioning (upper right panel of Fig. 7): the application of a spatial partitioning reduces the initial computation time by 30% to 50% and the penalisation phase 3 by up to 38%. Moreover, it reduces the overall time of more than 30%. The variance-covariance matrix computation was not affected. The time gain is negligible when the number of groups increase (i.e. $k \ge 4$);
- Model complexity (left panels of Fig. 7): when the number of basis functions *b* is reduced from 9 to 7, the computation time of all phases significantly decreases, independent of the approximation of the variance-covariance matrix. In particular, the penalised likelihood estimation decreases by up to 68% and the overall computation by 45%. When *b* further decreases to 5, the gain is less pronounced;
- Concerning the cross-validated model error, both MAE and RMSE are affected only by the model complexity (lower right panel of Fig. 7). Indeed, independent of the approximation of the covariance matrix or of the spatial partitioning, both MAE and RMSE decreased as the number of basis functions increased for all four criteria used to define the optimum $\lambda ^*_{min,PE}$.

In Fig. 8 we present the relationship between model complexity and the share of regressors removed with the adaptive LASSO. The plot clearly shows that when the number of basis functions is large, the overall proportion of zero coefficients increases up to 25% of the total. The graph on the right examines the excluded coefficients in detail and shows that the highest frequencies (corresponding to $\beta _6,\beta _7,\beta _8$ and $\beta _9$) are the most frequently removed by the algorithm. This is consistent with the $\hbox {NO}_2$ concentrations shown in Fig. 6. Indeed, since the response variable exhibites a very smooth intra-day pattern, the number of frequencies required to model the relationship with the covariates is low.

This result allows us to state that the adaptive LASSO algorithm proposed in this study can be a useful tool for identifying the most relevant frequencies as it is precise in its selection and implementable even in contexts with large data sets. If time computing time is not an issue, using a higher number of frequencies (e.g., $b=9$ in our case) provides better forecasting performance (i.e., lower RMSE and MAE) while avoiding an excessive number of non-zero coefficients.

### Penalised estimates

The above estimates show very limited variability across the models and exhibit weak sensitivity to the spatial partitioning and Hessian matrix approximations. As shown in the preceding section, the impact of our adaptive LASSO procedure is more pronounced as the number of fixed effects coefficients increases. Thus, we report and comment on the empirical results obtained considering the model with the lowest prediction error among those using $b=9$ basis functions. Specifically, we consider the case with an approximate variance-covariance matrix and without spatial partitioning.

**Fig. 9:**
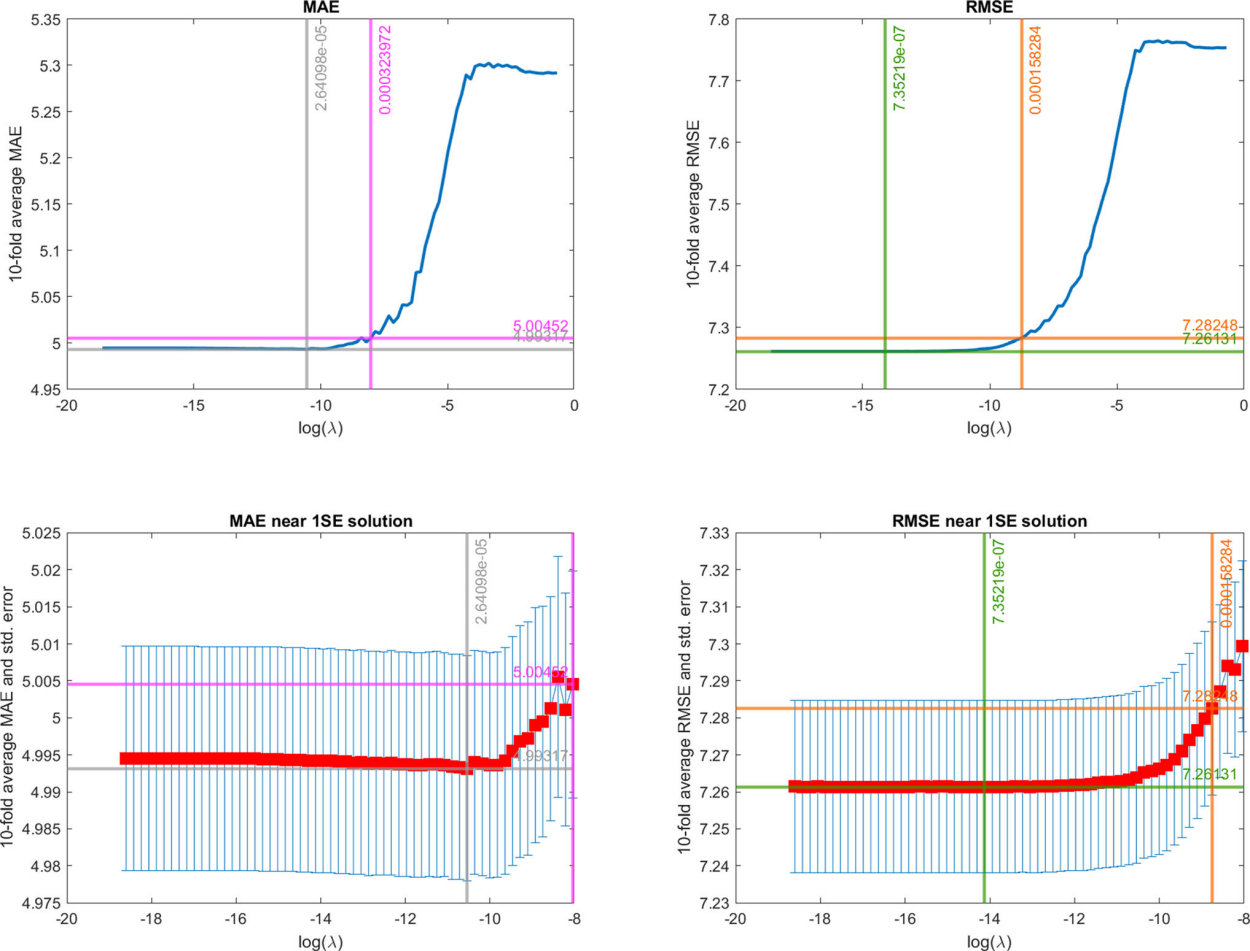
MAE against the logarithm of the penalty term $\lambda$ (left panels) and RMSE against the logarithm of the penalty term $\lambda$ (right panels). The horizontal lines represent the values of MAE and RMSE for key values of $log(\lambda )$, the optimal ($\lambda ^*_{min,RMSE}$ and $\lambda ^*_{min,MAE}$), 1-SE optimal values ($\lambda ^*_{1SE \ RMSE}$ and $\lambda ^*_{1SE \ MAE}$). The bottom panels are details near the optimum

**Fig. 10:**
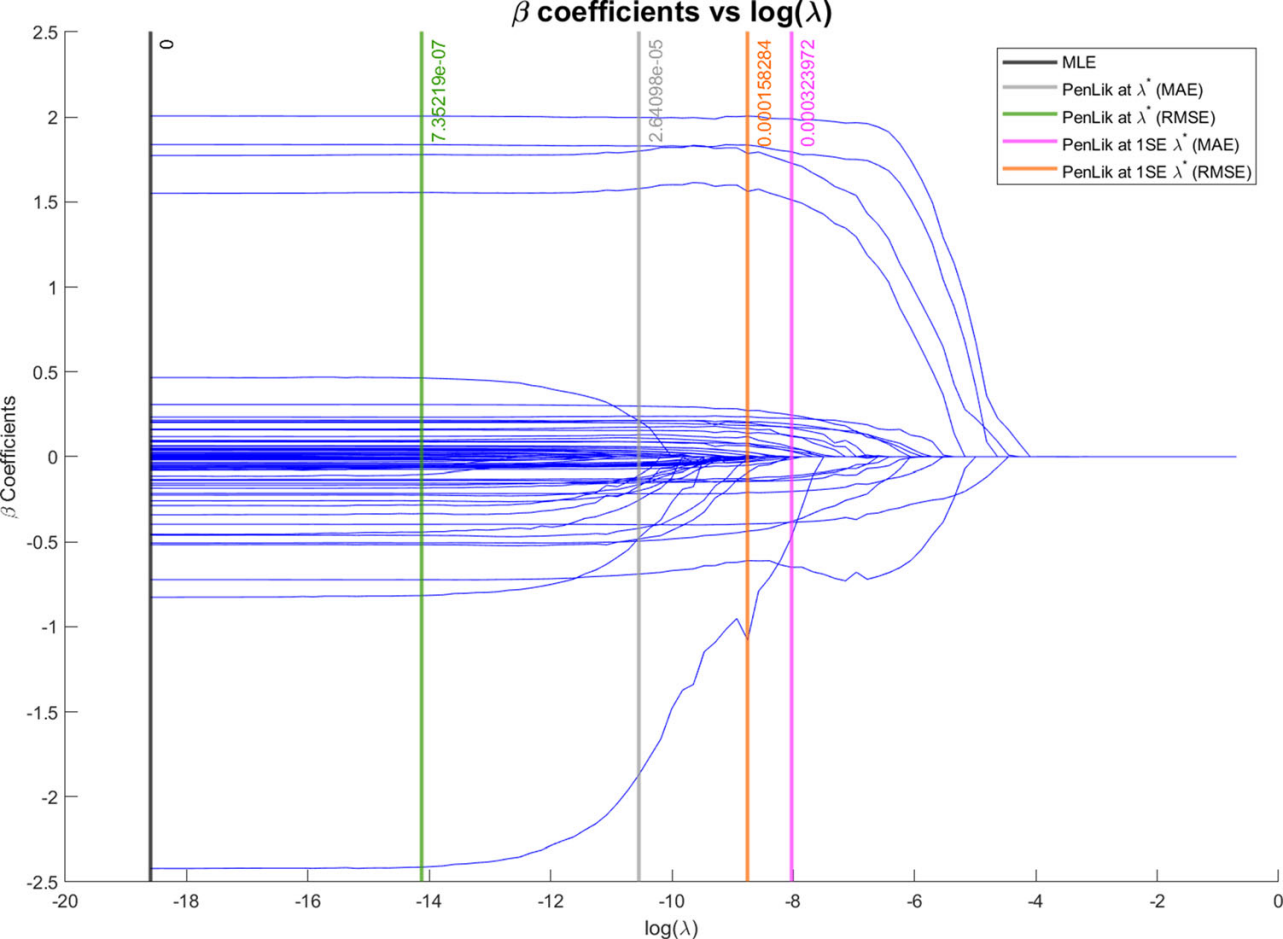
Functional coefficients against the logarithm of the penalty term $\lambda$. Vertical lines represent key values of $log(\lambda )$, i.e. unpenalized ($\lambda =0$), optimal ($\lambda ^*_{RMSE}$ and $\lambda ^*_{MAE}$), 1-SE rule ($\lambda ^*_{1\sigma ,RMSE}$ and $\lambda ^*_{1\sigma ,MAE}$)

In Fig. 9, we depict the behavior of the average cross-validation MAE (left panels) and RMSE (right panels) for increasing values of the penalty term $\lambda$. The computed RMSE falls between 7.26$\mu g/m^3$ (for small values of $\lambda$) and 7.75$\mu g/m^3$ (for large values of $\lambda$). These values are in the same scale as the observed standard deviation (shown in Fig. 6), but still below the minimum variability observed in the data (about 8.50 $\mu g/m^3$). By computing the ratio of the minimum MSE to the average intra-day variance we obtain the average proportion of variance unexplained by the model, equivalent to 32.34%. This percentage is consistent with the plot of the variance of measurement errors (Figure S1 in the Supplementary Information), whose average value is 28.60%. For large values of the penalty term (i.e., $log(\lambda ) > -4$) all the covariates are drop out. However, both one-standard-error-rules provide more parsimonious models with prediction errors not significantly different from the optimal ones. The estimated coefficients that correspond to the unrestricted model and the models associated with the four penalty terms considered are shown in Fig. 10.

**Fig. 11:**
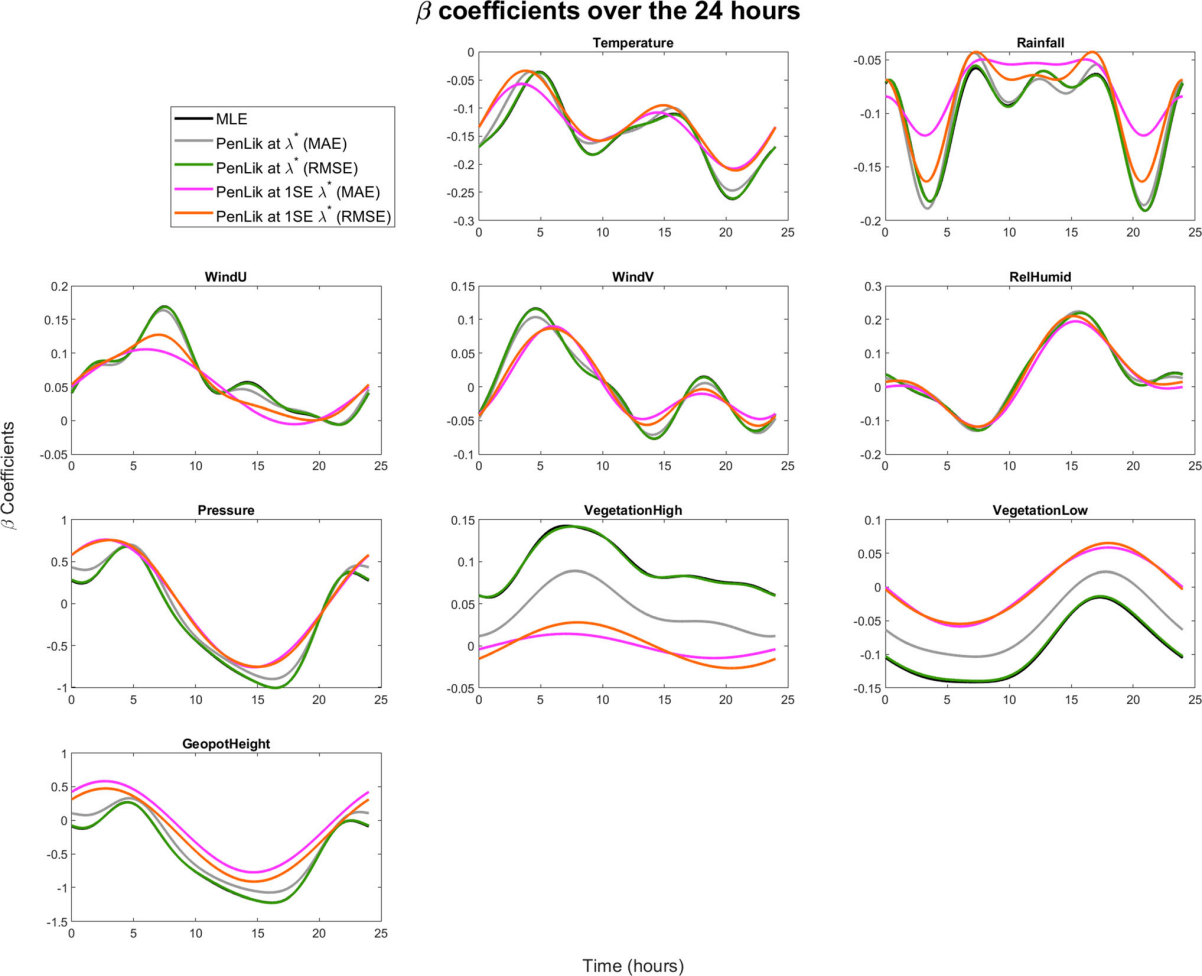
Estimated functional $\beta$ coefficients for differently selected optimal penalty parameters

In Fig. 11, we show the 24-hour estimated functional coefficients for each variable. The black lines correspond to the unpenalised MLE solution; the green lines to the optimal $\lambda$ w.r.t RMSE; the grey lines, to the optimal $\lambda$ w.r.t MAE; the orange lines, to the 1SE optimal $\lambda$ w.r.t RMSE; and the pink lines, to the 1SE optimal $\lambda$ w.r.t MAE. The estimated coefficients associated with temperature always exhibits negative values, particularly in the late afternoon and evening hours. The patterns obtained for different values of $\lambda$ did not show large discrepancies and tended to overlap throughout the day, leaving the overall dynamics unchanged during the day. However, the penalty seemed to mitigate the temperature effect at peak hours (10 a.m. and 8 p.m.). Rainfall shows a negative effect on the $\hbox {NO}_2$ concentrations, especially in the evening and just before dawn. In both moments, the effect reached the minimum peaks. Unlike the temperature, whose daily pattern varied slightly as the penalty increased, for higher values of $\lambda$, the precipitation diminished its effect and tended to flatten slightly towards 0. Considering the one-standard-error rule, between 7 a.m. and 5 p.m., the curve flattened to a constant negative value without being exactly 0. For the same $\lambda$ values, the two negative peak periods are greatly mitigated. These elements confirm the important role of temperature and rainfall in mitigating $\hbox {NO}_2$ concentrations, which is highlighted in literature (e.g., Fassò et al. 2022). Relative humidity presents some very interesting findings. First, its effect is null at around midnight, slightly negative at night before dawn and strongly positive in the daylight hours. Moreover, the penalisation appeared to produce no effect on the intra-day behaviour. This is consistent with the fact that whereas temperature and rainfall showed more complex patterns during the day, relative humidity already exhibits a simple pattern and did not need further smoothing.

Both the effects of atmospheric pressure and geopotential height (used as a proxy of elevation) depend on the moment of the day that was being considered. In both cases, the estimates show a positive effect at the start and at the end of the day and a negative effect in the afternoon. However, in the case of elevation, the functional coefficient in the early and late hours is very close to zero, and in the central hours, it deviates significantly from 0 regardless of the penalty used. For both variables, penalisation does not play a significant role, as the difference between penalised and non-penalised curves approaches 0 and the infra-daily dynamics appears to be stable.

Also, the U (eastward) and V (northward) components of wind show a time-varying behaviour across the day. In both cases, the effect on the $\hbox {NO}_2$ concentrations is positively estimated during the early stage of the day, especially between 5 a.m. and 10 a.m., and it strongly weakened in the afternoon and at night, reaching values very close to 0 between 3 p.m. and 8 p.m. However, the cleaning effect is limited to the early part of the day. The shrinkage effect induced by the penalisation algorithm is more pronounced in the eastward component than in the northward component. In fact, we noticed that the effect of the eastward component was strongly smoothed in the morning hours, and the coefficient was cancelled during the afternoon. The northward component, although also smoothed, showed a significantly positive effect in the early hours of the day.

Finally, we notice that penalisation generates a remarkable influence on the two land cover variables, that is the high vegetation and low vegetation indices. Both variables are heavily squeezed towards 0 even for the contained values of $\lambda$ until reaching zero for the values associated with the one-standard-error rule. Similar results are presented in Fassò et al. (2022), in which the effect of the same covariates on the $\hbox {NO}_2$, $\hbox {PM}_{10}$ and $\hbox {PM}_{2.5}$ concentrations in Lombardy is estimated to be close to 0, and thus not statistically significant, with the exception of the most urbanised areas.

To sum up, as expected, from Fig. 9 we see that from the predictive ability standpoint, penalised and non-penalised estimators are equivalent. However, Fig. 11 suggests that for some covariates the non-penalised intraday functional dynamics may be different from the penalised ones. In fact, for most of the covariates the smoothing increases moving from the MLE to the one-standard-error solution. In this regard, the functional coefficient of the rainfall shrinks close to zero in the middle of the day, whereas the vegetation variables lose much of their importance shrinking close to zero for the large part of the day. So, compared to the MLE and $\lambda ^*_{min}$ solutions, the one-standard-error estimate leads to a simpler and more interpretable model. This holds both for the RMSE and the MAE.

## Conclusions and future developments

In this paper, we introduced an adaptive LASSO estimator for the so called functional hidden dynamic geostatistical models (f-HDGM). This new estimation approach based on penalised maximum-likelihood estimation can be used to efficiently estimate the relevant model coefficients and shrink to zero the irrelevant ones, while taking into account spatiotemporal correlation and cross-correlation among predictors. In addition to the case in which all the coefficients of the splines basis associated with a certain covariate are set to zero, it is possible that only some of them are shrunk to zero. Indeed, the algorithm can be successfully applied to identify the relevant part of a functional coefficient across its functional domain.

From a computational perspective, we showed that the estimation can be efficiently implemented as a local quadratic approximation around the maximum of the log-likelihood function. To find this maximum, the EM algorithm can be used (see Wang et al. 2021). Then, a BFGS quasi-Newton iterative method can be used to optimise the penalised function.

We analysed the performance of this estimation procedure through a Monte Carlo simulation study based on three settings with increasing level of complexity and representative of common applied contexts. To be precise, we considered settings where only parts of the functional coefficients had zero effects and where the regressors were cross-correlated and driven by spatiotemporal dynamics, as is often observed in geostatistical applications. The results of the simulations show that from the pure prediction ability perspective, penalised estimates are equivalent to maximum likelihood estimates. However, from an inferential standpoint, the one-standard-error estimates lead to models much more parsimonious and easier to interpret than the MLE estimates. As expected, having simulated in a Gaussian context, MAE and RMSE give almost equivalent results. Finally, the estimates produced by the one-standard-error rule enjoy an oracle property in the sense that for the null coefficients we detect the true zero value.

Furthermore, we applied have the penalisation algorithm to an empirical example of air quality assessment using hourly $NO_2$ concentrations observed in Lombardy, Italy. Like the simulations, the empirical results reveal that from the pure prediction capability perspective, the penalised estimates are equivalent to the maximum likelihood estimates. While the solutions of the one-standard-error rule for RMSE and MAE are very similar, the minimum solution of the MAE suggests a higher degree of penalisation for the coefficients. In terms of inference, the one-standard-error estimates of the coefficients associated with different weather variables are very smoothed and in some cases almost zero. We also provided an extended study of the scalability of the algorithm when applied to real world data. In particular, we showed that even with high model complexity, the computation time (both of the penalised likelihood and overall) can greatly benefit from approximations in model estimation, leaving performance essentially unaffected.

This paper focused on model selection in functional data contexts, performed using an adaptive LASSO penalisation algorithm. However, further extensions can be pursued. Our proposal may be useful in several environmental policy assessment contexts, such as agricultural policies (Fass‘o, A., Rodeschini, J., Moro, A.F., Shaboviq, Q., Maranzano, P., Cameletti, M., Otto, P. 2023), air quality assessment (Fassò et al. 2022), and energy policies (Yuan et al. 2018). Indeed, the smoothness of the estimated functional coefficients can also be of high interest in such applications because too large a number of spline bases leads to over-fitting and non-smooth estimated effects. From the methodological perspective, a further penalty term based on the integrated second derivatives could counter these effects. Thus, an elastic net structure that includes the smoothness penalty and an adaptive LASSO penalty is a very interesting topic for future research. Eventually, using the results of Simon and Tibshirani (2012), the standardised group-LASSO estimator could be extended to spatiotemporal functional models by optimising a penalized likelihood function with quadratic approximation and by assuming that the spline basis functions associated with each covariate are a group.

## Supplementary Information

Below is the link to the electronic supplementary material.

Supplementary file 1 (pdf 2400 KB)

## Data Availability

All the results presented in this paper can be reproduced using MATLAB and R software. Data and codes for both the simulation and application results are available at the following GitHub web page: https://github.com/PaoloMaranzano/PM_PO_AF_AdaptiveLASSO_SERRA2023.git

## Footnotes

1 Given the Markovian dynamics with respect to the time (Eq. 4) and the presence of the Gaussian Process with covariance matrix $\Gamma$ (Eq. 5), it is easily demonstrated that the variance of the spatiotemporal effect $\omega _{st}(h)$ (Eq. 3) depends: (a) linearly on the marginal variances $v_1,\ldots ,v_p$; (b) non-linearly (hyperbole) on the temporal autocorrelation coefficient *g*; (3) non-linearly (exponential) on the range $\theta$. Figure S3 in the Supplementary Information shows how the variability of $n=10$ simulated random effects and the total variability evolves as the temporal autocorrelation, marginal variance and range change.

2 Following Ramsay (2005) (Section 3.4), a set of periodic Fourier basis functions is defined as $B_0(h)=1$, $B_{2K_a-1}=sin(hK_a\frac{2\pi }{24})$, and $B_{2K_a}=cos(hK_a\frac{2\pi }{24})$
