# Supplementary material for: Adaptive LASSO estimation for functional hidden dynamic geostatistical models
Source: Stoch Environ Res Risk Assess. 2023 May 17:1–23. Online ahead of print. doi: 10.1007/s00477-023-02466-5 (PMC10189237; doi:10.1007/s00477-023-02466-5)
Supplement: Supplementary file 1 — (pdf 2400 KB) [file 477_2023_2466_MOESM1_ESM.pdf]

Supplementary Information

Adaptive LASSO estimation for functional hidden dynamic geostatistical models

Paolo Maranzano[a,b], Philipp Otto[c], Alessandro Fassò[d]

[a] *University of Milano-Bicocca, Piazza dell'Ateneno Nuovo 1, Milano, 20126, MI, Italy*
[b] *Fondazione Eni Enrico Mattei (FEEM), Corso Magenta 63, Milano, 20123, MI, Italy*
[c] *Leibniz University Hannover, Appelstrasse 9a, Hannover, 30167, Lower Saxony, Germany*
[d] *University of Bergamo, Viale Marconi 5, Dalmine, 24044, BG, Italy*

Extended results about the scalability of the algorithm applied to air quality in Lombardy

VarCov	Spatial partition	Basis (b)	DSTEM time (Minutes)	VarCov time (Minutes)	PenLik time (Minutes)	Total time (Minutes)	min RMSE	1-SE min RMSE	min MAE	1-SE min MAE
**Approx**	**k = 1**	**9**	**84.94**	**39.98**	**156.83**	**281.75**	**7.26**	**7.28**	**4.99**	**5.00**
Exact	k = 1	9	83.45	126.10	155.82	365.37	7.26	7.28	4.99	5.01
Approx	k = 2	9	61.79	38.76	97.80	198.35	7.27	7.29	5.00	5.00
Exact	k = 2	9	61.14	127.86	98.13	287.13	7.27	7.28	5.00	5.00
Approx	k = 3	9	43.61	38.62	102.28	184.52	7.28	7.30	5.00	5.01
Approx	k = 4	9	21.63	38.99	105.50	166.12	7.28	7.30	5.00	5.01
Approx	k = 5	9	21.48	37.34	103.58	162.40	7.28	7.30	5.00	5.01
Approx	k = 2	7	49.40	24.07	53.90	127.37	7.46	7.48	5.10	5.10
Exact	k = 2	7	49.08	75.22	53.98	178.28	7.46	7.48	5.10	5.10
Approx	k = 1	7	83.37	23.52	49.48	156.37	7.46	7.48	5.10	5.10
Exact	k = 1	7	80.26	71.38	49.90	201.53	7.46	7.48	5.10	5.10
Approx	k = 1	5	48.97	11.28	33.22	93.47	7.73	7.76	5.25	5.26
Exact	k = 1	5	49.15	43.15	32.72	125.02	7.73	7.76	5.25	5.26
Approx	k = 2	5	31.15	13.72	35.05	79.92	7.74	7.75	5.25	5.26
Approx	k = 3	5	22.42	12.63	36.87	71.92	7.74	7.76	5.25	5.26
Approx	k = 4	5	13.20	11.50	37.58	62.28	7.74	7.75	5.25	5.26
Exact	k = 2	5	31.19	42.21	35.10	108.50	7.74	7.75	5.25	5.26

Table S1: Summary of the empirical models.

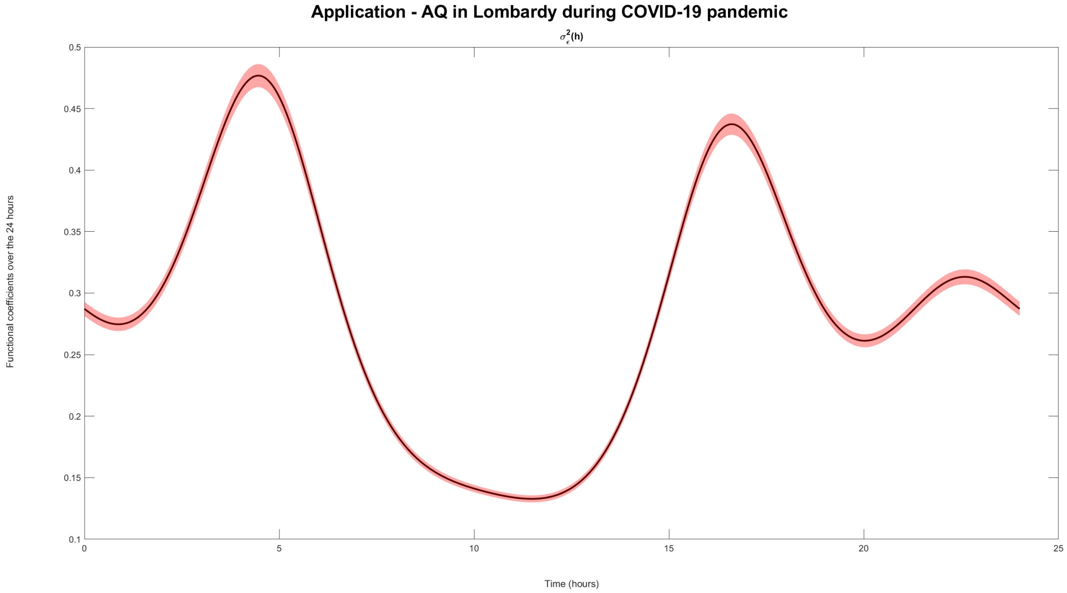

Figure S1: Estimated 24-hours functional model error variance (σ_ε^2) for the optimal model. Solid line represents an unbiased point estimate of the parameters, whereas the range of the red shaded areas reflects their 95% confidence intervals. The values are bounded between 13% (minimum) and 47% (maximum), with an average value of 28.60%.

Note: Having both the response variable and the regressors standardized, the values range from 0% to 100%, thus they can be interpreted as the portion (%) of unexplained NO_2 variance along the day.

Functional box-plots of the time-varying covariates

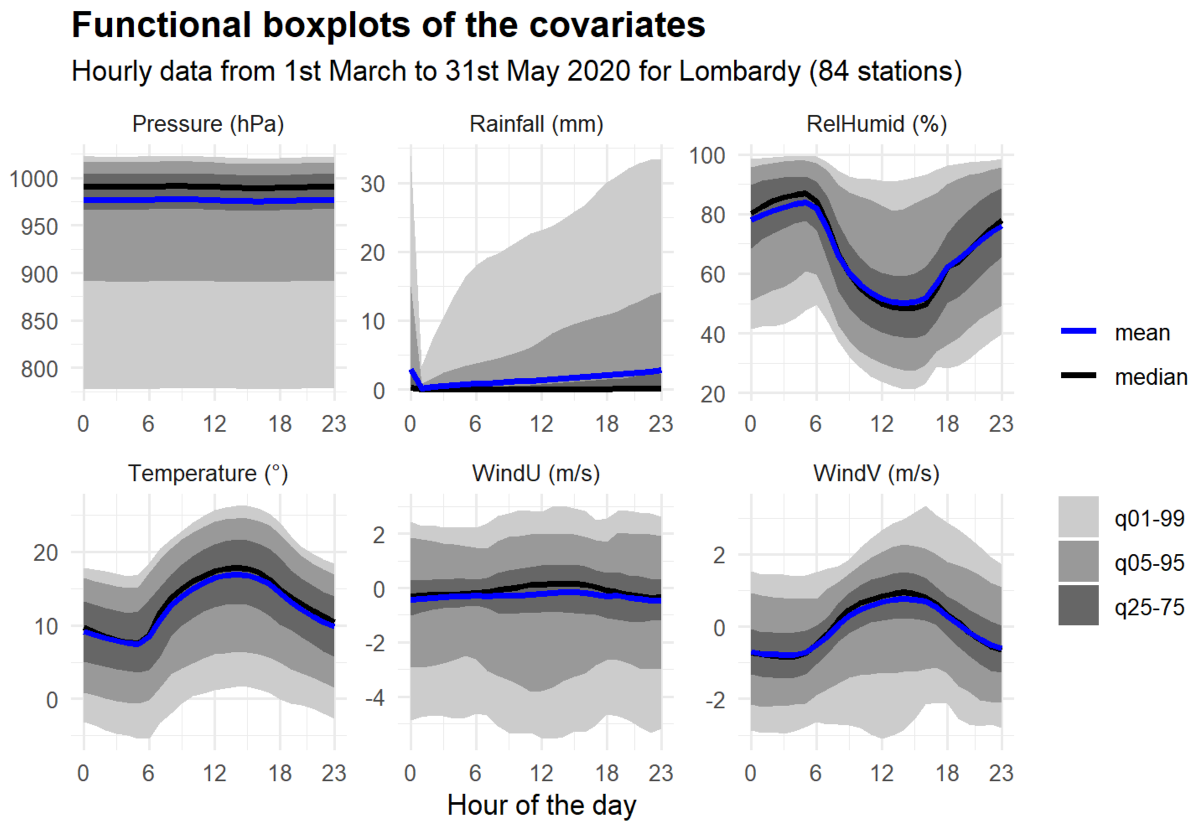

Figure S2: Functional box-plots for each time-varying covariate used in the application: pressure, relative humidity, temperature, rainfall, eastward (U) component of wind and northward (V) component of wind.

Simulated reference values for the MSE, RMSE and MAE of each setting

		**MSE**	**RMSE**	**MAE**
Setting I	min	0.9999	1.0000	0.7979
		(0.0004)	(0.0002)	(0.0002)
	max	2.7257	1.6510	1.2724
		(0.0013)	(0.0004)	(0.0003)
Setting II	min	0.9989	0.9995	0.7975
		(0.0004)	(0.0002)	(0.0002)
	max	2.7238	1.6504	1.2719
		(0.0013)	(0.0004)	(0.0003)
Setting III	min	0.9996	0.9998	(0.7977
		(0.0004)	(0.0002)	(0.0002)
	max	5.1389	2.2669	1.6956
		(0.0022)	(0.0005)	(0.0003)

Table S2: Simulated ($n = 100$ simulations) reference values for each setting. Reported values are the average across simulations of the minimum (*min*) and maximum (*max*) MSE, RMSE and MAE. Values under parenthesis are the standard error of the mean computed across the simulations.

Effect of spatiotemporal parameters on the random effect variance and on the total variance of simulated data

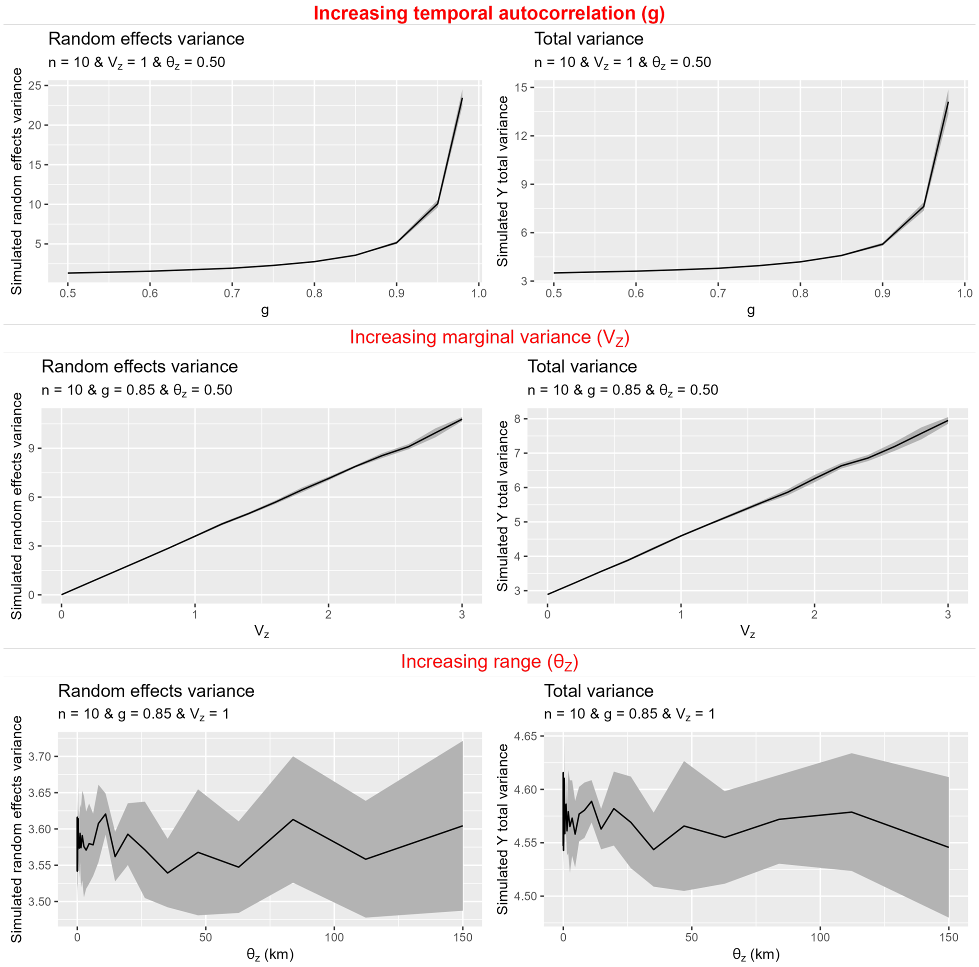

Figure S3: Effect of spatio-temporal parameters (v_Z,g,θ) on the random effect variance (left) and on the total variance of simulated data (right). Solid black line represents the unbiased point estimate of the mean, whereas shaded grey areas are the 95% confidence interval computed using $n = 10$ simulations.

Setting I: spatio-temporal uncorrelated observations and uncorrelated covariates

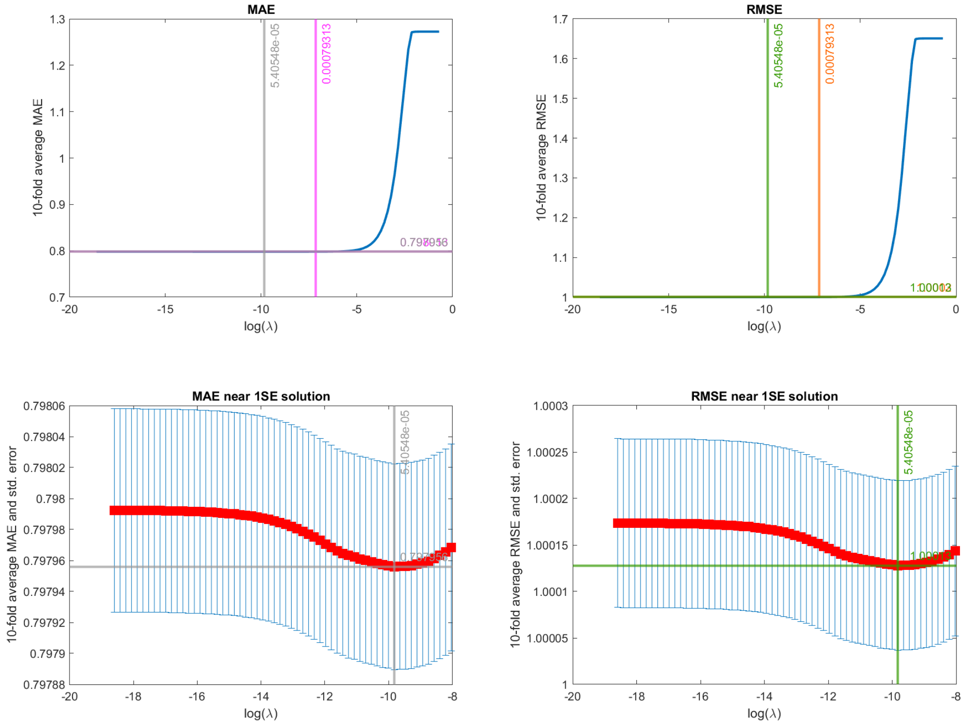

Figure S4: RMSE and MAE for different values of λ in Setting I. Top panels: full λ range. Bottom panels: near-optimum λ range. Left panels: MAE. Right panels: RMSE. The vertical and horizontal lines correspond to the considered selection rules (grey: λ^*_{MAE}; pink: $\lambda^*_{1SE\ MAE}$; black: λ^*_{RMSE}; orange: $\lambda^*_{1SE\ RMSE}$).

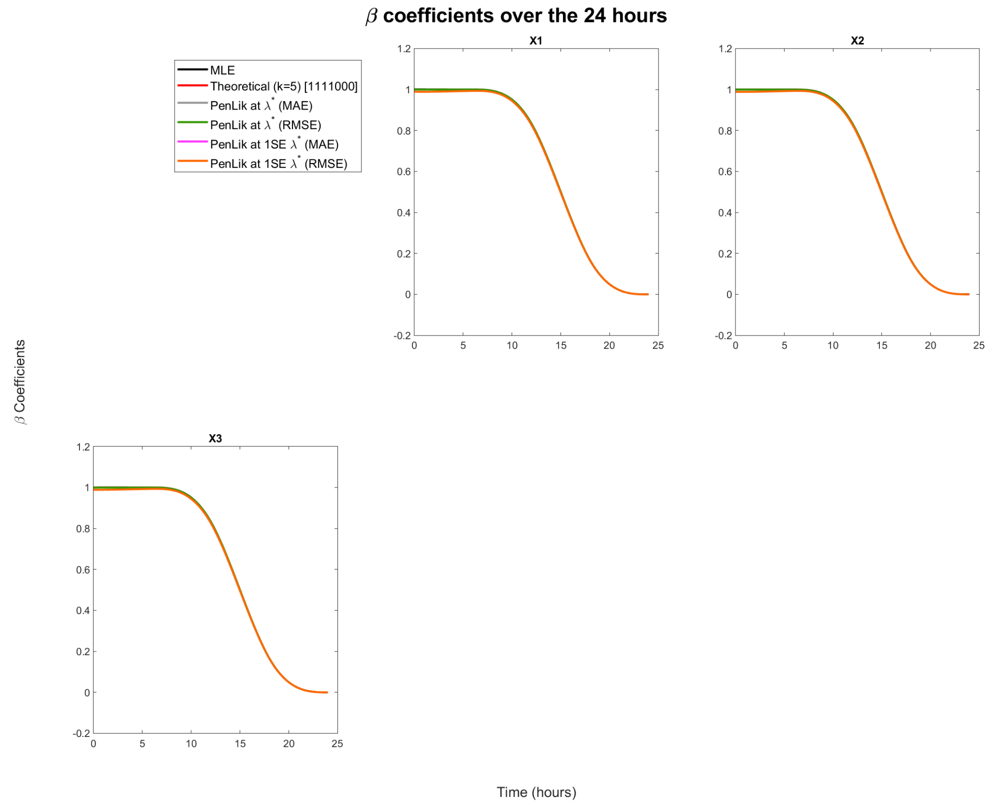

Figure S5: Average estimated functional coefficients of each variable at several optimal λ values for Setting I.

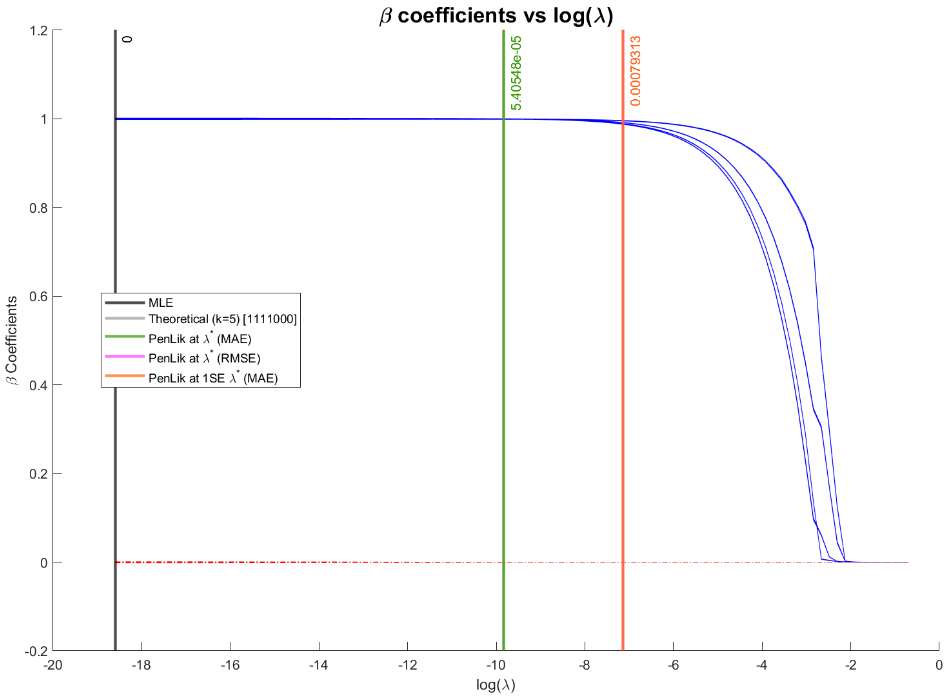

Figure S6: Average estimated coefficients for different values of λ in Setting I. The positive coefficients are drawn in blue, while the zero coefficients are depicted by the red dashed lines.

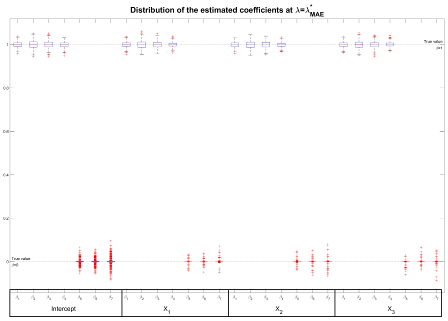

(a) $\lambda_{min\ MAE}$

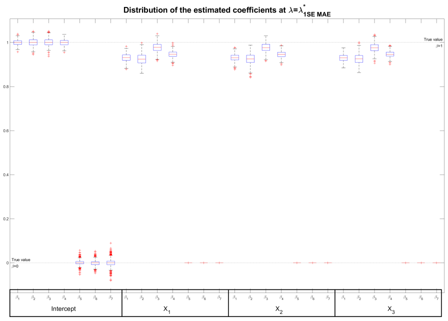

(b) $\lambda_{1SE\ MAE}$

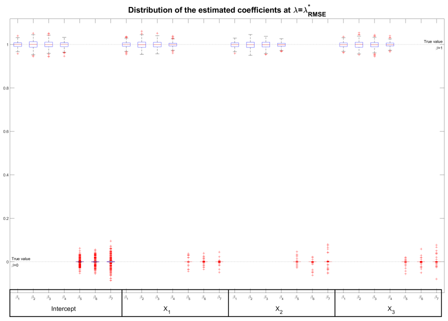

(c) $\lambda_{min\ RMSE}$

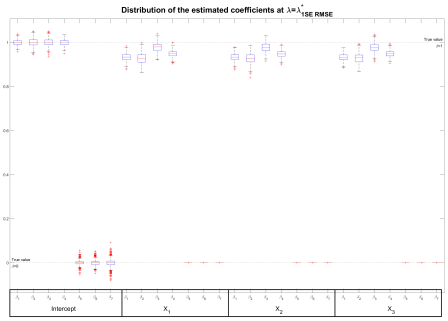

(d) $\lambda_{1SE\ RMSE}$

Figure S7: Box plot of the estimated coefficients across 500 simulations at $\lambda^* = \lambda_{min\ MAE}$ (upper left panel), at $\lambda^* = \lambda_{min\ RMSE}$ (lower left panel), at $\lambda^* = \lambda_{1SE\ MAE}$ (upper right panel) and at $\lambda^* = \lambda_{1SE\ RMSE}$ (lower right panel) for Setting I.

Table S3: Average value and root mean squared error (RMSE) of each β coefficient at several optimal λ positions across $k = 500$ simulations

		MLE		$\lambda_{min\ RMSE}$		$\lambda_{1-SE\ RMSE}$		$\lambda_{min\ MAE}$		$\lambda_{1-SE\ MAE}$	
Variable	Coefficient	Mean	RMSE	Mean	RMSE	Mean	RMSE	Mean	RMSE	Mean	RMSE
$Intercept$	β_1	0.9998	0.0124	0.9998	0.0124	0.9997	0.0123	0.9999	0.0124	0.9997	0.0123
$Intercept$	β_2	1.0000	0.0183	1	0.0179	1.0001	0.0181	0.9999	0.018	1.0001	0.0181
$Intercept$	β_3	1.0008	0.0181	1.0008	0.017	1.0007	0.0174	1.0009	0.017	1.0008	0.0173
$Intercept$	β_4	0.9995	0.0157	0.9995	0.0132	0.9997	0.014	0.9994	0.0131	0.9996	0.014
$Intercept$	β_5	0.0005	0.0192	0.0005	0.0121	0.0003	0.0139	0.0007	0.0121	0.0004	0.0141
$Intercept$	β_6	-0.0008	0.0219	-0.0005	0.0127	-0.0003	0.0152	-0.0007	0.0126	-0.0005	0.015
$Intercept$	β_7	0.0009	0.0301	0.0003	0.0197	0	0.0233	0.0007	0.02	0.0002	0.023
X_1	β_1	1.0008	0.0129	0.9999	0.0129	0.9337	0.0687	0.9998	0.0129	0.931	0.0712
X_1	β_2	1.0005	0.0185	0.9994	0.018	0.9273	0.0766	0.9992	0.0181	0.9245	0.0795
X_1	β_3	0.9999	0.0182	0.9998	0.0159	0.9781	0.0302	0.9997	0.016	0.9773	0.0309
X_1	β_4	1.0007	0.0161	0.9996	0.0105	0.9483	0.0536	0.9996	0.0108	0.9462	0.0556
X_1	β_5	-0.0006	0.0202	0	0.0045	0	0	-0.0001	0.0053	0	0
X_1	β_6	-0.0004	0.0225	0	0.0037	0	0	0	0.0048	0	0
X_1	β_7	0.0005	0.0283	-0.0001	0.0041	0	0	0.0002	0.0067	0	0
X_2	β_1	1.0003	0.0123	0.9993	0.0123	0.9331	0.069	0.9992	0.0123	0.9305	0.0715
X_2	β_2	1.0002	0.018	0.9993	0.0174	0.9273	0.0765	0.9992	0.0176	0.9244	0.0796
X_2	β_3	1.0001	0.0177	0.9996	0.0155	0.978	0.03	0.9995	0.0156	0.9772	0.0306
X_2	β_4	0.9998	0.0153	0.9994	0.0106	0.948	0.0539	0.9994	0.0109	0.9459	0.056
X_2	β_5	0.0007	0.0184	0.0001	0.0037	0	0	0	0.005	0	0
X_2	β_6	-0.0005	0.0218	-0.0004	0.0051	0	0	-0.0001	0.0061	0	0
X_2	β_7	0.0007	0.0301	0.0002	0.0084	0	0	-0.0002	0.0093	0	0
X_3	β_1	1.0000	0.0121	0.9991	0.0121	0.9329	0.0692	0.999	0.0122	0.9303	0.0717
X_3	β_2	1.0011	0.0171	0.9999	0.0166	0.9281	0.0753	0.9998	0.0166	0.9253	0.0781
X_3	β_3	0.9985	0.0171	0.9987	0.0151	0.9768	0.0304	0.9985	0.0152	0.9759	0.0313
X_3	β_4	1.0017	0.0159	1.0003	0.0104	0.949	0.053	1.0004	0.0107	0.947	0.0548
X_3	β_5	-0.0015	0.019	-0.0002	0.0039	0	0	-0.0005	0.0053	0	0
X_3	β_6	0.001	0.022	-0.0001	0.005	0	0	0.0002	0.0055	0	0
X_3	β_7	-0.0006	0.0301	0.0003	0.0075	0	0	-0.0002	0.0071	0	0

Note: Mean is computed as the average across simulations of the β values on the full sample (without splitting into CV fold); *RMSE* is computed as the average across simulation of the squared-root distances between the average β values and the true coefficients.

Setting II: spatio-temporal correlated observations and uncorrelated covariates

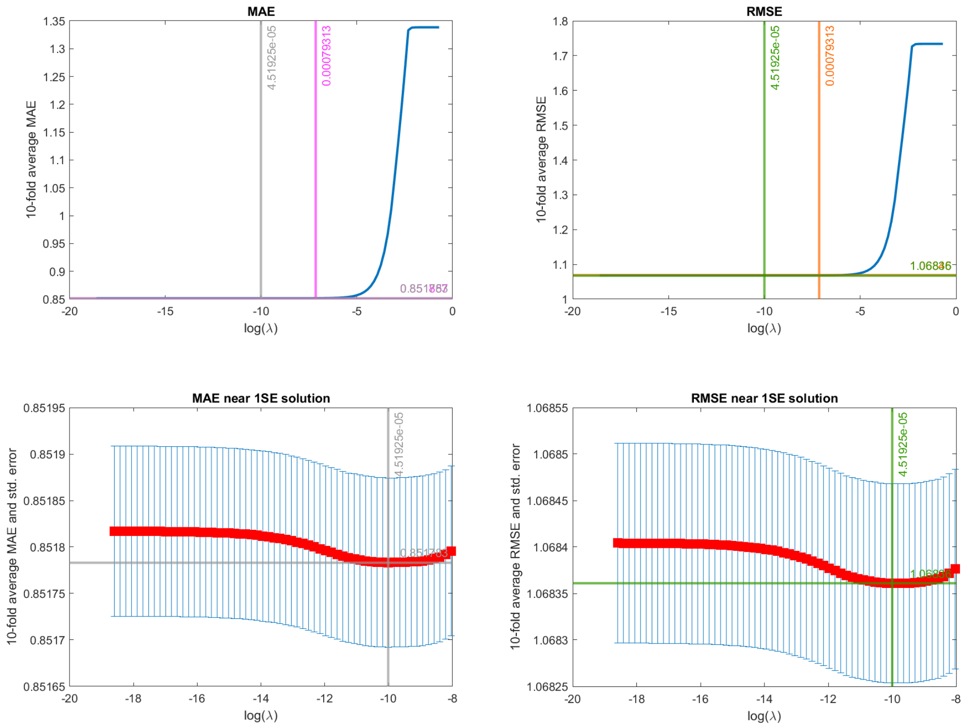

Figure S8: RMSE and MAE for different values of λ in Setting II. Top panels: full λ range. Bottom panels: near-optimum λ range. Left panels: MAE. Right panels: RMSE. The vertical and horizontal lines correspond to the considered selection rules (grey: λ^*_{MAE}; pink: $\lambda^*_{1SE\ MAE}$; black: λ^*_{RMSE}; orange: $\lambda^*_{1SE\ RMSE}$).

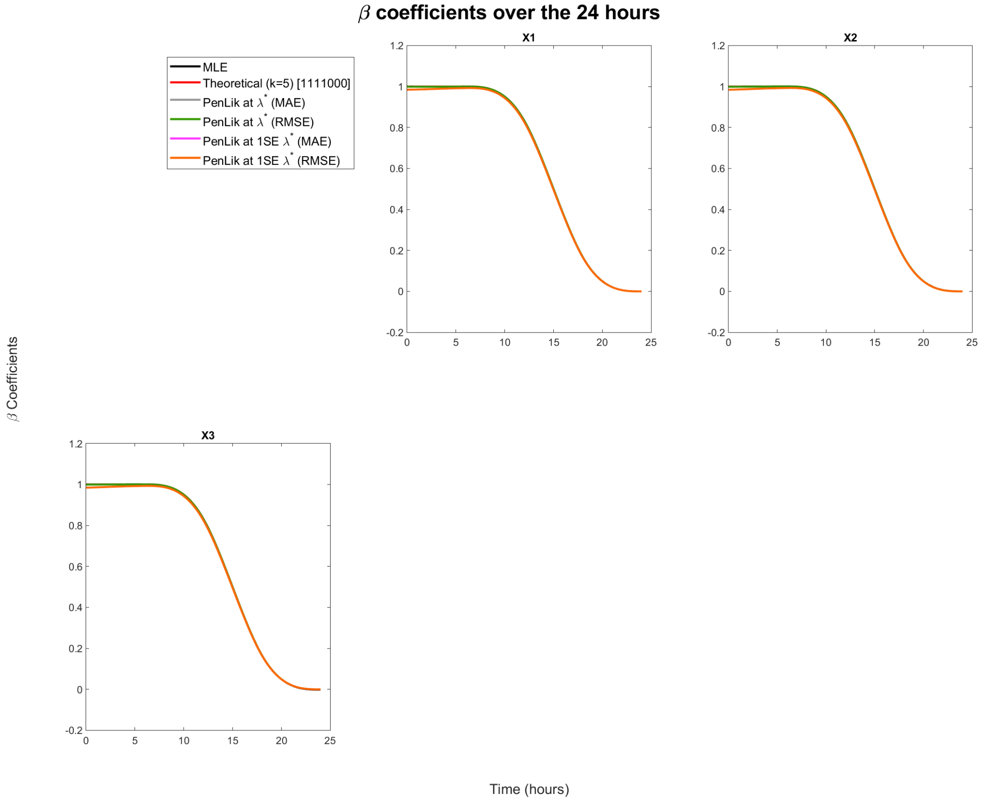

Figure S9: Average estimated functional coefficients of each variable at several optimal λ values for Setting II.

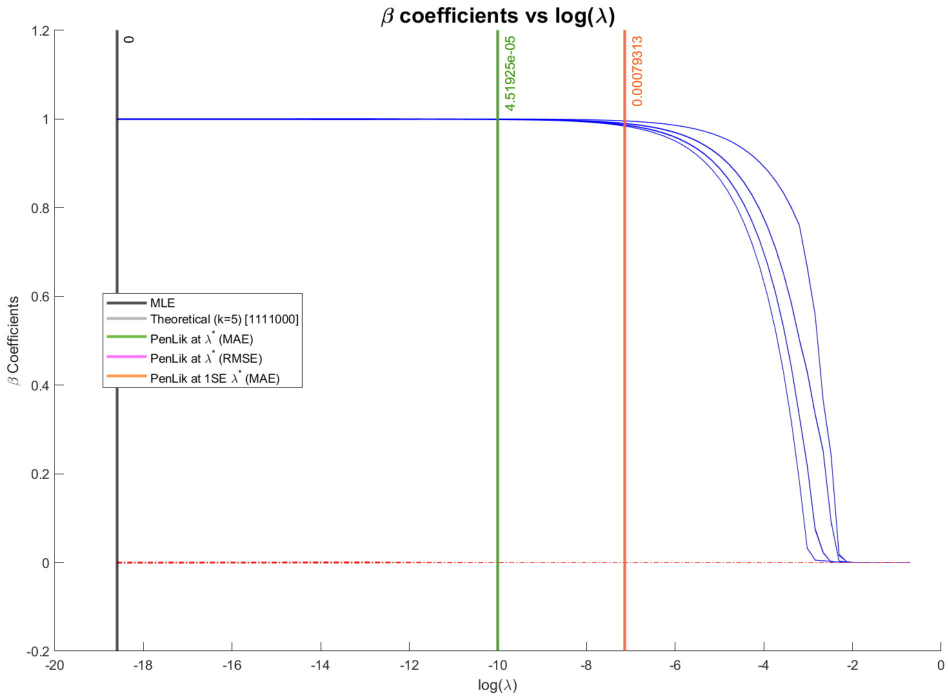

Figure S10: Average estimated coefficients for different values of λ in Setting II. The positive coefficients are drawn in blue, while the zero coefficients are depicted by the red dashed lines.

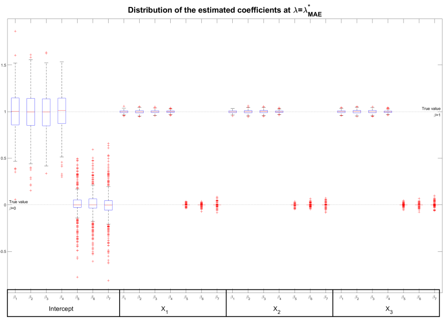

(a) $\lambda_{min\ MAE}$

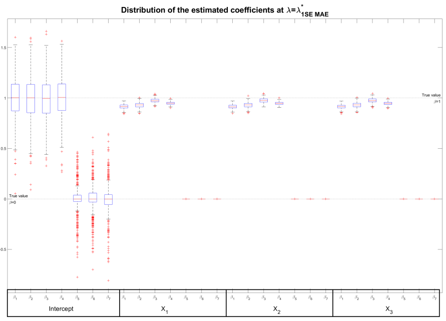

(b) $\lambda_{1-SE\ MAE}$

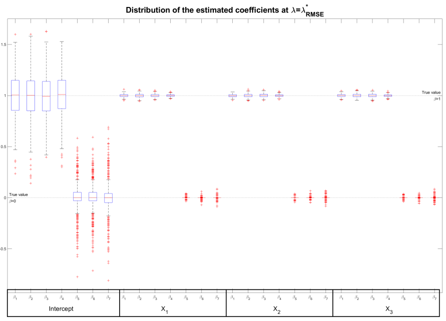

(c) $\lambda_{min\ RMSE}$

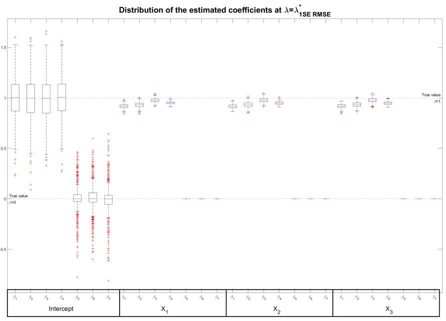

(d) $\lambda_{1-SE\ RMSE}$

Figure S11: Box plot of the estimated coefficients across 500 simulations at $\lambda^* = \lambda_{min\ MAE}$ (upper left panel), at $\lambda^* = \lambda_{min\ RMSE}$ (lower left panel), at $\lambda^* = \lambda_{1SE\ MAE}$ (upper right panel) and at $\lambda^* = \lambda_{1SE\ RMSE}$ (lower right panel) for Setting II.

Table S4: Average value and root mean squared error (RMSE) of each β coefficient at several optimal λ positions across $k = 500$ simulations

		MLE		$\lambda_{min\ RMSE}$		$\lambda_{1-SE\ RMSE}$		$\lambda_{min\ MAE}$		$\lambda_{1-SE\ MAE}$	
Variable	Coefficient	Mean	RMSE	Mean	RMSE	Mean	RMSE	Mean	RMSE	Mean	RMSE
$Intercept$	β_1	0.9968	0.2141	0.9947	0.2206	0.9948	0.218	0.9958	0.2233	0.9953	0.2199
$Intercept$	β_2	0.995	0.2121	0.9928	0.2262	0.9949	0.216	0.9905	0.2254	0.9938	0.2169
$Intercept$	β_3	0.9914	0.2078	0.9888	0.2154	0.9899	0.2143	0.9882	0.2168	0.9916	0.2126
$Intercept$	β_4	1.0051	0.1967	1.0044	0.2039	1.0039	0.2003	1.0016	0.2046	1.0039	0.2011
$Intercept$	β_5	0.0048	0.1993	0.0008	0.163	0.0015	0.1582	0.0011	0.1655	-0.0012	0.1567
$Intercept$	β_6	0.0036	0.1995	-0.0007	0.1578	0.0026	0.1553	0.0021	0.1624	0.0009	0.1552
$Intercept$	β_7	-0.0124	0.2108	-0.0031	0.1742	-0.0047	0.1698	-0.0066	0.1759	-0.0029	0.1676
X_1	β_1	1.0003	0.0151	0.9994	0.0151	0.9182	0.0845	0.9991	0.0151	0.915	0.0877
X_1	β_2	0.9992	0.0188	0.9984	0.0186	0.9319	0.0727	0.9983	0.0187	0.9292	0.0754
X_1	β_3	1.0004	0.018	1.0001	0.0158	0.9763	0.0316	1	0.016	0.9753	0.0324
X_1	β_4	1.0002	0.0161	0.9997	0.0111	0.9504	0.0515	0.9996	0.0115	0.9485	0.0535
X_1	β_5	0.0004	0.0206	0.0002	0.0059	0	0	0.0001	0.0068	0	0
X_1	β_6	-0.0001	0.0251	-0.0005	0.007	0	0	-0.0006	0.0077	0	0
X_1	β_7	0.0002	0.0324	-0.0001	0.0099	0	0	0.0004	0.0114	0	0
X_2	β_1	0.9999	0.0142	0.999	0.0142	0.9179	0.0844	0.9987	0.0143	0.9147	0.0877
X_2	β_2	1.0000	0.0189	0.9993	0.0183	0.9327	0.072	0.9991	0.0184	0.9302	0.0744
X_2	β_3	1.0007	0.0183	1.0004	0.0163	0.9768	0.0312	1.0003	0.0164	0.9758	0.0323
X_2	β_4	0.9996	0.0158	0.9991	0.0115	0.9495	0.0527	0.999	0.0117	0.9476	0.0546
X_2	β_5	-0.0002	0.0195	-0.0002	0.0058	0	0	-0.0003	0.0066	0	0
X_2	β_6	0.0005	0.0242	0.0001	0.0067	0	0	-0.0001	0.008	0	0
X_2	β_7	0.0001	0.0336	0	0.0104	0	0	0	0.0112	0	0
X_3	β_1	1.0002	0.0144	0.9992	0.0144	0.9182	0.0841	0.9989	0.0146	0.915	0.0875
X_3	β_2	1.0003	0.0188	0.9997	0.0181	0.9331	0.0714	0.9995	0.0182	0.9305	0.074
X_3	β_3	1.001	0.0196	1.0004	0.0172	0.9768	0.0322	1.0003	0.0174	0.9758	0.0331
X_3	β_4	0.9989	0.0169	0.9987	0.0121	0.9492	0.053	0.9986	0.0127	0.9473	0.055
X_3	β_5	0.0008	0.0205	0.0001	0.0054	0	0	0.0001	0.0077	0	0
X_3	β_6	-0.0011	0.0243	-0.0002	0.0062	0	0	-0.0003	0.0092	0	0
X_3	β_7	-0.0017	0.034	0.0001	0.0115	0	0	0.0009	0.0141	0	0

Note: Mean is computed as the average across simulations of the β values on the full sample (without splitting into CV fold); *RMSE* is computed as the average across simulation of the squared-root distances between the average β values and the true coefficients.

Setting III: spatio-temporal correlated observations and correlated covariates

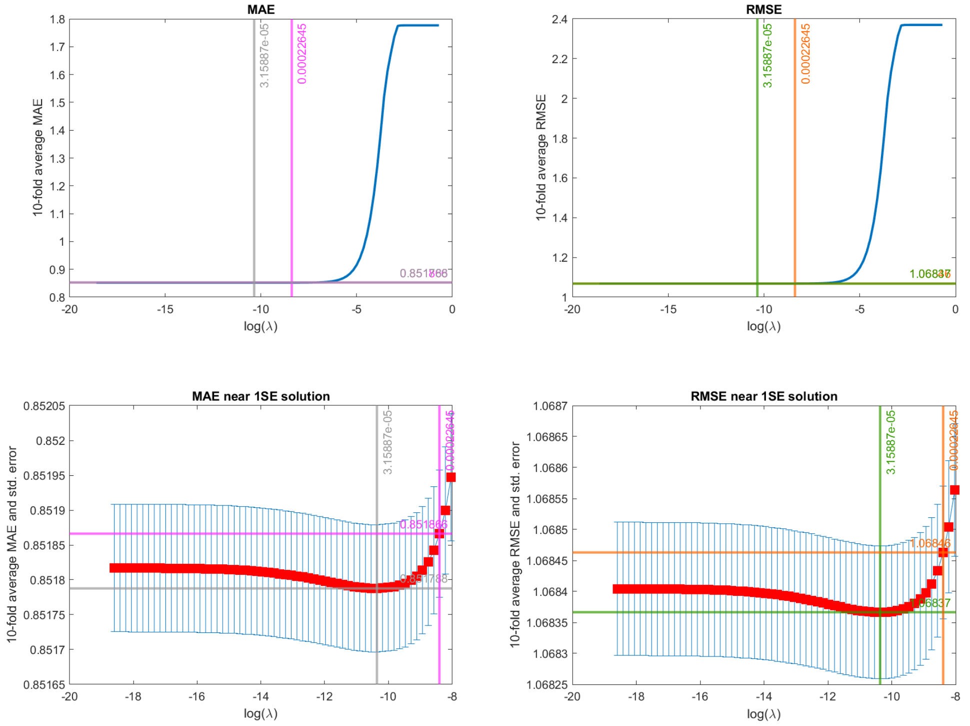

Figure S12: RMSE and MAE for different values of λ in Setting III. Top panels: full λ range. Bottom panels: near-optimum λ range. Left panels: MAE. Right panels: RMSE. The vertical and horizontal lines correspond to the considered selection rules (grey: λ^*_{MAE}; pink: $\lambda^*_{1SE\ MAE}$; black: λ^*_{RMSE}; orange: $\lambda^*_{1SE\ RMSE}$).

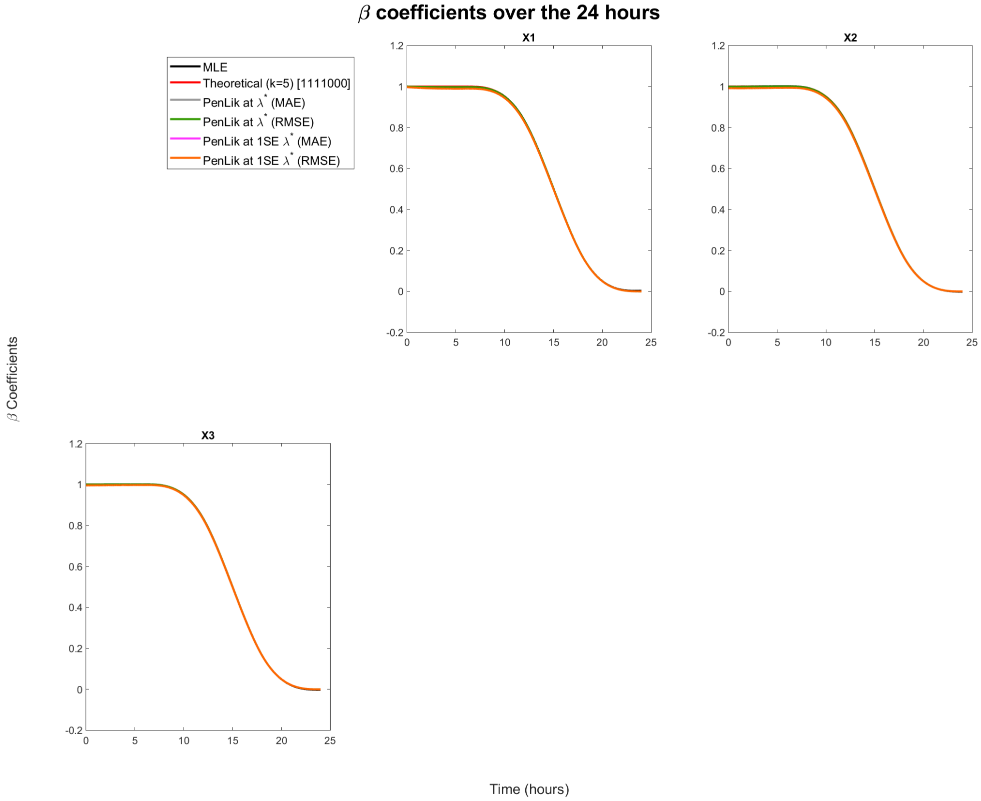

Figure S13: Average estimated functional coefficients of each variable at several optimal λ values for Setting III.

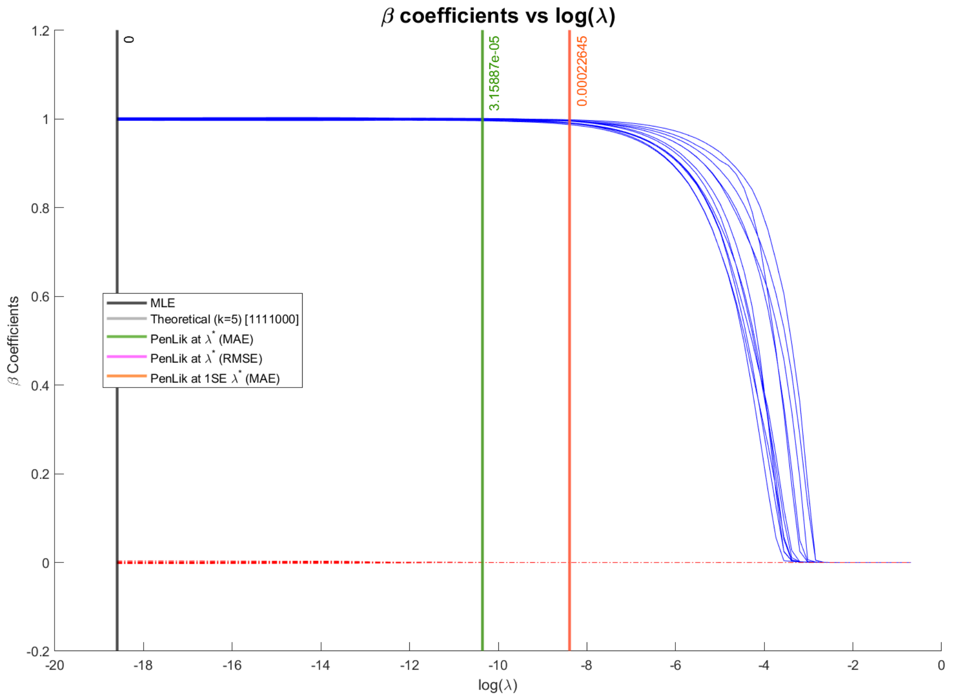

Figure S14: Average estimated coefficients for different values of λ in Setting III. The positive coefficients are drawn in blue, while the zero coefficients are depicted by the red dashed lines.

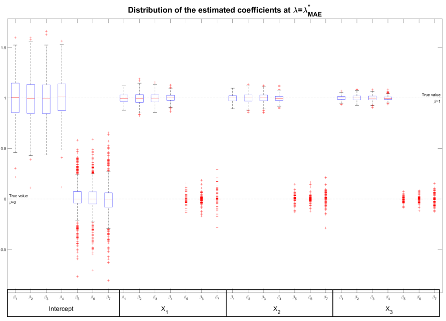

(a) $\lambda_{min\ MAE}$

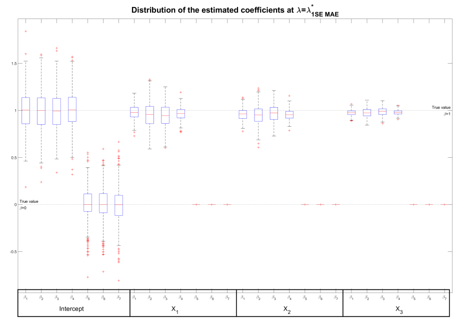

(b) $\lambda_{1-SE\ MAE}$

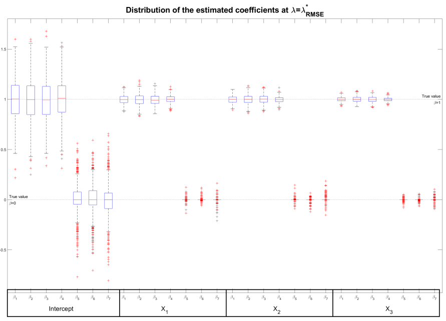

(c) $\lambda_{min\ RMSE}$

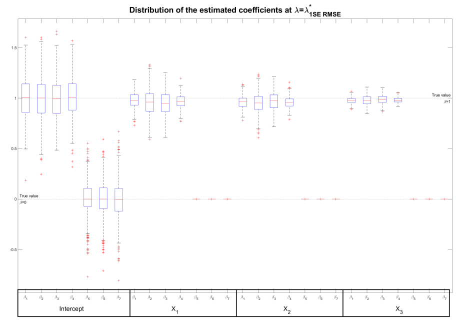

(d) $\lambda_{1-SE\ RMSE}$

Figure S15: Box plot of the estimated coefficients across 500 simulations at $\lambda^* = \lambda_{min\ MAE}$ (upper left panel), at $\lambda^* = \lambda_{min\ RMSE}$ (lower left panel), at $\lambda^* = \lambda_{1SE\ MAE}$ (upper right panel) and at $\lambda^* = \lambda_{1SE\ RMSE}$ (lower right panel) for Setting III.

Table S5: Average value and root mean squared error (RMSE) of each β coefficient at several optimal λ positions across $k = 500$ simulations

		MLE		$\lambda_{min\ RMSE}$		$\lambda_{1-SE\ RMSE}$		$\lambda_{min\ MAE}$		$\lambda_{1-SE\ MAE}$	
Variable	Coefficient	Mean	RMSE	Mean	RMSE	Mean	RMSE	Mean	RMSE	Mean	RMSE
Intercept	β_1	0.9968	0.2141	0.995	0.2166	0.9976	0.2142	0.9959	0.2177	0.9987	0.2167
Intercept	β_2	0.995	0.2121	0.9924	0.2145	0.996	0.2124	0.9922	0.217	0.9954	0.2118
Intercept	β_3	0.9914	0.2078	0.9903	0.2115	0.9923	0.2063	0.9904	0.211	0.992	0.2079
Intercept	β_4	1.0051	0.1967	1.003	0.1982	1.0064	0.1968	1.0033	0.1995	1.0064	0.1973
Intercept	β_5	0.0048	0.1993	0.0045	0.1794	0.005	0.1886	0.0041	0.18	0.0039	0.1903
Intercept	β_6	0.0036	0.1995	0.002	0.1787	0.0015	0.1912	0.0011	0.178	0.0016	0.1914
Intercept	β_7	-0.0124	0.2108	-0.009	0.1907	-0.0072	0.2034	-0.0101	0.1865	-0.007	0.2024
X_1	β_1	1.0001	0.0447	0.9999	0.0451	0.9832	0.0787	1.0001	0.0452	0.9828	0.0797
X_1	β_2	0.9986	0.0576	0.9972	0.0561	0.9567	0.1336	0.997	0.0561	0.9552	0.1363
X_1	β_3	0.9969	0.0589	0.9965	0.0523	0.9475	0.1284	0.9968	0.0527	0.9464	0.1307
X_1	β_4	1.0032	0.0523	1.0015	0.0368	0.9666	0.0735	1.0011	0.0383	0.9654	0.0751
X_1	β_5	-0.0009	0.0623	0.0004	0.0137	0	0	0.0011	0.0204	0	0
X_1	β_6	0.0011	0.0739	0.0012	0.0127	0	0	-0.0003	0.0221	0	0
X_1	β_7	0.0034	0.0956	-0.0013	0.0217	0	0	0.0006	0.0331	0	0
X_2	β_1	1.0000	0.0359	0.999	0.0362	0.9584	0.0733	0.9988	0.0363	0.9572	0.0747
X_2	β_2	1.0003	0.0474	0.9993	0.0464	0.9501	0.1123	0.9995	0.0464	0.949	0.1138
X_2	β_3	1.0027	0.0471	1.0018	0.0423	0.9698	0.0958	1.0015	0.0425	0.9687	0.0977
X_2	β_4	0.998	0.0418	0.9973	0.0298	0.9576	0.0671	0.9976	0.0311	0.9565	0.0682
X_2	β_5	0.0005	0.0511	0.0003	0.0135	0	0	-0.0004	0.0179	0	0
X_2	β_6	0.0000	0.0614	-0.0014	0.0134	0	0	0	0.0196	0	0
X_2	β_7	-0.0016	0.0824	0.0018	0.0222	0	0	-0.0002	0.0273	0	0
X_3	β_1	1.0003	0.0222	0.9996	0.0223	0.9758	0.0393	0.9996	0.0223	0.9751	0.04
X_3	β_2	1.0004	0.029	1.0002	0.0282	0.9758	0.0531	1.0003	0.0282	0.9752	0.0539
X_3	β_3	1.0016	0.0302	1.0006	0.027	0.9883	0.0456	1.0005	0.0272	0.9879	0.0464
X_3	β_4	0.9983	0.0261	0.9987	0.019	0.9794	0.0329	0.9988	0.0198	0.9789	0.0335
X_3	β_5	0.0013	0.0315	-0.0002	0.0095	0	0	-0.0005	0.013	0	0
X_3	β_6	-0.0016	0.0374	-0.0001	0.0086	0	0	0.0001	0.0146	0	0
X_3	β_7	-0.0026	0.0524	-0.0009	0.0161	0	0	-0.0004	0.0234	0	0

Note: Mean is computed as the average across simulations of the β values on the full sample (without splitting into CV fold); *RMSE* is computed as the average across simulation of the squared-root distances between the average β values and the true coefficients.
